# Luopan Mountain Pig Bone Marrow Mesenchymal Stem Cells Promote Liver Regeneration in D-Galactosamine-Induced Acute Liver Failure Rats by Regulating the PTEN-PI3K/Akt/mTOR Pathway

**DOI:** 10.3390/biology14101363

**Published:** 2025-10-05

**Authors:** Minjuan Li, Zhongfa Wang, Xingxing Yan, Yanchen Liu, Yunan He, Bianying Zhang, Weijun Guan

**Affiliations:** 1 Department of Animal Genetic Resources, Institute of Animal Science, Chinese Academy of Agricultural Sciences, Beijing 100193, China; 17806280540@163.com (M.L.); 19722725957@163.com (Z.W.);; 2 Department of Animal Genetics, Breeding and Reproduction, College of Animal Science, Shanxi Agricultural University, Jinzhong 030801, China; 3 Department of Public Physical Education, Luoyang Normal University, Luoyang 471934, China

**Keywords:** acute liver failure, bone marrow-derived mesenchymal stem cells, Luopan Mountain pig, PI3K/Akt/mTOR pathway, PTEN, liver regeneration, D-galactosamine

## Abstract

**Simple Summary:**

Acute liver failure is a serious condition in which the liver stops working. Treatment options for this condition are limited, as there are not enough donor organs for transplants and the body often rejects transplanted organs. In this study, we investigated a potential new treatment using stem cells from a special breed of pig. We aimed to assess whether these cells could help to repair the damaged liver in rats with induced liver failure. Our results showed that injecting these pig stem cells into the rats greatly improved their survival. The treatment helped the liver to heal by reducing tissue damage, improving function, and encouraging the organ to regenerate. We also discovered that the cells work by activating a specific repair pathway in the liver. This research is a first step toward a new treatment for liver failure that could help to save human lives by providing an alternative to scarce human organ transplants in the future.

**Abstract:**

Treatment for acute liver failure (ALF) is constrained by shortages of liver transplant donors and immune rejection. Porcine bone marrow mesenchymal stem cells (pBMSCs) demonstrate clinical potential in xenotransplantation due to their abundant availability, low immunogenicity, and strong proliferative activity. This study is the first to investigate the reparative effects and mechanisms of pBMSCs derived from Luopan Mountain pigs in a D-galactosamine (D-GalN)-induced ALF rat model. The results demonstrated that tail-vein transplantation of pBMSCs significantly improved survival rates in ALF rats; reduced serum ALT, AST, and TBIL levels; enhanced hepatic glycogen metabolism; and mitigated histopathological liver damage. Additionally, pBMSC transplantation upregulated serum HGF, IGF-1, and VEGF levels while inhibiting hepatocyte apoptosis. Mechanistic studies indicate that pBMSCs promote liver function recovery and regeneration by activating the PI3K/Akt/mTOR signaling pathway and suppressing its key negative regulator, PTEN, by regulating the expression of key genes involved in inflammation, fibrosis, proliferation, and apoptosis. This study provides crucial experimental evidence for the use of pBMSCs in treating acute liver failure (ALF) and lays the groundwork for its clinical translation in the field of xenotransplantation.

## 1. Introduction

ALF is a critical clinical syndrome characterized by rapid hepatocyte necrosis, coagulation dysfunction, and hepatic encephalopathy [1,2,3]. D-GalN induces hepatocyte necrosis by depleting UTP and disrupting UDP-glucose metabolism, making it widely used in animal models of ALF [4]. At present, liver transplantation remains the only effective treatment; however, donor shortages, postoperative complications, and high costs severely limit its clinical application [5,6].

In recent years, BMSCs have gained attention as a promising treatment for ALF, owing to their capacity for multilineage differentiation, low immunogenicity, and paracrine properties [7,8]. Research indicates [9] that hBMSCs overexpressing CCR2 had enhanced homing capacity in ALF mouse models, significantly suppressing inflammatory infiltration and promoting liver regeneration. However, the clinical application of hBMSCs still faces challenges, including donor ethical controversies, limited in vitro expansion capacity, and potential xenogeneic immune barriers [10]. pMSCs exhibit unique advantages in xenotransplantation due to their high physiological similarity to human stem cells, wide availability, and ease of standardized preparation [11]. Extensive research has demonstrated the promising therapeutic potential of pMSCs across various disease models. In liver injury repair, Bama miniature pADMSCs effectively alleviated CCl_4_-induced acute liver failure in mice [12]. In bone regeneration, pBMSCs combined with biomaterial scaffolds successfully promoted bone defect repair [13]. Furthermore, studies in kidney transplantation models have confirmed that infusing pADMSCs into ischemic-injured porcine kidneys did not induce adverse reactions and had no significant impact on renal function or structure, validating their biological safety [14]. Despite the potential of allogeneic MSCs in regenerative medicine, their long-term safety remains a concern. Studies have indicated that single or multiple injections of high-dose hUC-MSCs or hBMSCs did not induce tumors in mice [15], and a 26-week tumorigenicity assessment of hUC-MSCs was also negative [16]. However, MSCs subjected to immortalization or genetic modification retain potential tumorigenic risks, necessitating monitoring of chromosomal stability [17]. Although MSCs possess multipotent differentiation capabilities, the risk of ectopic differentiation is low. No significant abnormal differentiation or chromosomal aberrations were observed in hUC-MSCs after long-term cryopreservation or in non-human primates [18].

The PI3K/Akt/mTOR axis is critically involved in hepatic repair processes through its regulatory effects on cellular proliferation, apoptotic activity, and metabolic homeostasis [19,20]. Studies have indicated that, in acute liver injury models, activation of this pathway can mitigate inflammatory responses and improve liver function by modulating macrophage M1/M2 polarization [21]. In CCl_4_-induced rat models of liver fibrosis, inhibiting pathway phosphorylation blocked hepatic stellate cell activation, thereby reducing fibrosis severity [22]; in models of hepatic ischemia–reperfusion injury, blackberry extract improved liver function by promoting pathway phosphorylation and downregulating pro-apoptotic protein expression [23]. PTEN is a key negative regulator of the PI3K/Akt/mTOR signaling pathway, and suppression of its expression also significantly promotes liver regeneration [24,25]. However, whether pBMSCs repair acute liver failure by modulating the PTEN-PI3K/Akt/mTOR signaling axis remains unclear.

The therapeutic potential and mechanism of action of pBMSCs derived from Luopan Mountain pigs—a rare pig breed endemic to China [26]—in ALF models have not yet been systematically investigated. Based on this, Luoshan pig pBMSCs were employed for the first time in this study using a rat model of ALF induced by D-GalN, with the aim of systematically elucidating the core regulatory mechanisms of the PTEN-PI3K/Akt/mTOR signaling axis in pBMSC-mediated liver regeneration. The findings provide crucial experimental evidence for xenogeneic stem cell therapy in ALF and lay the groundwork for its clinical use.

## 2. Materials and Methods

### 2.1. Isolation and Culture of pBMSCs

Under aseptic conditions, 7-day-old Luopan Mountain pig femurs were harvested, and muscle and connective tissue were removed. Both epiphyses were trimmed, and the medullary cavity was flushed with H-DMEM medium containing 13% FBS. The wash solution was collected and centrifuged, and the supernatant was removed. The cell suspension was then transferred to 6-well plates at a density of 1 × 10^4^ cells per well. Cell culture was performed in a 37 °C, 5% CO_2_ incubator. Cells were subcultured upon attaining 90–95% confluency. Purified bone marrow mesenchymal stem cells were obtained after three passages.

### 2.2. Immunofluorescence Detection of pBMSCs

P4 pBMSCs were seeded in 6-well plates. Cells at 60% confluency were fixed in 4% paraformaldehyde for 20 min, permeabilized with 0.25% Triton X-100 for 15 min, and blocked with goat serum for 30 min. Primary antibodies against CD29 (1:200, bs-0486R, Bioss, Woburn, MA, USA), CD34 (1:300, bs-0646R, Bioss), CD44 (1:300, bs-2780R, Bioss), CD73 (1:200, bs-23233R, Bioss), CD90 (1:200, bs-49172R, Bioss), and CD105 (1:200, bs-34063R, Bioss) were added separately. Cells were incubated overnight with primary antibodies at 4 °C in the dark, washed, then treated with FITC-conjugated goat anti-rabbit secondary antibody (1:500, bs-0295G, Bioss) for 1 h at room temperature in the dark. The secondary antibody was removed, and nuclei were stained with DAPI for 20 min in the dark. Finally, the cells were rinsed with PBS and visualized under a confocal microscope.

### 2.3. pBMSCs Multidirectional Differentiation Ability Detection

P4 pBMSCs were plated into 24-well plates. When reaching 70% confluence, the medium was replaced with osteogenic, adipogenic, or chondrogenic induction medium, which was refreshed every 3 days for differentiation. Morphological changes were observed under an inverted microscope during differentiation: Osteogenic group-After mineralized nodules formed, calcium deposition was detected by alizarin red staining. Adipogenic group-Once lipid droplets appeared, they were identified via Oil Red O staining. Chondrogenic group-Proteoglycan content was assessed via alizarin blue staining after formation of chondrocyte-like clusters.

### 2.4. Construction of a Rat Model of Acute Liver Failure

This study was approved by the Animal Welfare and Ethics Committee of the Institute of Animal Science, Chinese Academy of Agricultural Sciences (Beijing) (Approval No.: IAS2025-128). All of our experiments were conducted in strict accordance with the ARRIVE guidelines. A total of 45 6-week-old SPF male SD rats (body weight 190–210 g, purchased from the China National Institute for Food and Drug Control) were acclimated for 7 days and then randomly divided into three groups (*n* = 15): the Sham group (intraperitoneal injection of equal-volume saline), D-GalN group (intraperitoneal injection of 600 mg/kg D-GalN), or pBMSC transplantation group (D-GalN + pBMSCs; 2 h after intraperitoneal injection of equivalent D-GalN dose, tail-vein injection of 2 × 10^6^ CM-Dil-labeled pBMSCs). Rats in each group were anesthetized at 6, 12, 24, 48, and 72 h post pBMSC transplantation. Blood samples and liver tissue were collected for biochemical and histopathological analysis.

#### 2.4.1. Serum Biochemical Indicator Testing

Serum levels of liver function markers ALT, AST, TBIL, and ALB were measured using a fully automated biochemical analyzer (BS360S, Shenzhen, China) following the manufacturer’s strict operating protocols.

#### 2.4.2. Serum Cytokine Testing

Serum levels of IL-6, IL-8, IL-1β, TNF-α, HGF, IGF-1, and VEGF were measured using ELISA kits (Jianglai Bio, Shanghai, China). All experimental protocols were performed in full compliance with the manufacturer’s guidelines.

#### 2.4.3. Liver Tissue Pathology Testing

Liver tissue was fixed in 4% paraformaldehyde, then processed into paraffin-embedded blocks and sliced into sections, and processed with xylene dewaxing and graded ethanol hydration. Hepatic necrosis and inflammatory infiltration were assessed using HE staining (Pinofly, Shenzhen, China). Collagen deposition was analyzed via Masson’s trichrome staining (Pinofly, Shenzhen, China) to evaluate the severity of fibrosis. Pathological changes were observed and recorded with light microscopy.

#### 2.4.4. Detection of Apoptosis in Liver Tissue Cells

Following dewaxing and rehydration of liver tissue sections, apoptosis was detected using the TUNEL assay kit (Sevier Bio, Wuhan, China), Samples were processed as per the manufacturer’s guidelines and observed under a fluorescence microscope. The apoptosis-positive cell rate (apoptotic cells/total cells × 100%) was calculated by randomly selecting 5 fields of view via ImageJ (Version 1.51).

#### 2.4.5. Liver Tissue Glycogen Staining

After dewaxing and rehydration of liver tissue sections, staining was performed according to the PAS staining kit (Pinofly, Shenzhen, China) protocol. Microscopic examination revealed purple-red granular glycogen deposits following hematoxylin counterstaining.

#### 2.4.6. Immunohistochemical Testing

The liver tissue sections were blocked with 5% BSA after dewaxing. Rabbit anti-Ki67 (1:500, ABB00008, Huilan, Shenzhen, China) and PCNA primary antibody (1:1000, 10205-2-AP, PTG, Shenzhen, China) were sequentially added and cultured overnight at 4 °C. After rinsing with PBS, HRP-labeled goat anti-rabbit secondary antibody (1:500, PN0046, Pinotfly, Shenzhen, China) was applied and incubated at room temperature for one hour. Staining with DAB, counterstaining with hematoxylin, dehydration, and mounting were then performed. Using light microscopy, 3 non-overlapping fields of view were randomly selected. The ImageJ 1.51K software was employed to compute the proportion of the positive area (positive area/total field area × 100%) at a uniform threshold.

#### 2.4.7. Real-Time Fluorescent Quantitative PCR Analysis

Total RNA was extracted from liver tissue using the Trizol method and reverse transcribed into cDNA. Amplification was performed on a quantitative PCR instrument using SYBR Green qPCR Master Mix (Q311-03, Vazyme, Nanjing, China). GAPDH served as the housekeeping gene. The relative mRNA expression levels of *TNF-α*, *IL-6*, *IL-1β*, *TGF-β1*, *Acta2*, *Bax*, *Bcl-2*, *PCNA*, and *Myc* were calculated using the 2^−ΔΔCt^ method. Primer sequences are listed in Table S1.

#### 2.4.8. Western Blot Analysis

Total liver tissue proteins were extracted using RIPA lysis buffer (P1005, Pinnacle, Jiangsu, China) containing protease and phosphatase inhibitors. Equal amounts of proteins were separated via SDS-PAGE and transferred to PVDF membranes. These were then incubated with rabbit anti-GAPDH (1:15,000, AB0037, Abways, Shanghai, China), p-Akt (1:2000, CY6569, Abways, Shanghai, China), primary antibodies—Bcl-2 (1:3000, 26593-1-AP, Proteintech, Rosemont, IL, USA), Cleaved Caspase-3 (1:2000, 25128-1-AP, Proteintech), PTEN (1:2000, CY5231, Abways, Shanghai, China), mouse anti-p-mTOR (1:10,000, 67778-1-Ig, Proteintech, IL, USA), and Bax (1:10,000, 60267-1-Ig, Proteintech, IL, USA)—were incubated at 4 °C overnight. Next, cells were treated with an HRP-labeled goat anti-rabbit/mouse secondary antibody (1:15,000, G1213, Servicebio, Wuhan, China) for 1 h at room temperature. After ECL development, chemiluminescent imaging was performed, and ImageJ was used to calculate relative expression levels by assessing the grayscale ratio of target proteins to GAPDH.

### 2.5. Statistical Analysis

All results are expressed as mean ± SD and were analyzed statistically using GraphPad Prism version 10.1.2. One-way ANOVA was used for multi-group comparisons, two-way ANOVA for bivariate analyses, and subjected to Tukey’s post hoc analysis for multiple comparisons. * *p* < 0.05 indicates a significant difference, and ** *p* < 0.01 indicates a highly significant difference.

## 3. Results

### 3.1. Biological Characteristics of Luopan Mountain Pig pBMSCs

**Figure 1:**
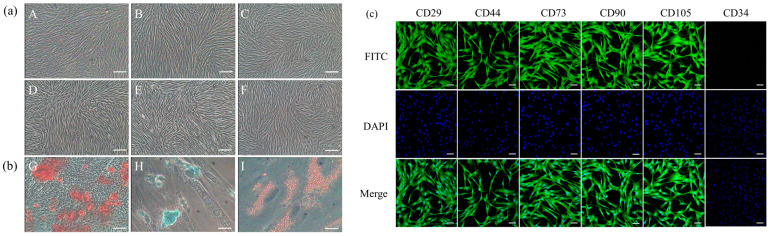
Biological characteristics of Luopan Mountain pig pBMSCs. (**a**) Morphology of cells at different passages: P0 (A), P1 (B), P4 (C), P8 (D), P12 (E), and P16 (F) (scale bar = 100 μm). (**b**) Multilineage differentiation potential assessed by specific staining. Representative images of Alizarin Red S (red, G), Alcian Blue (blue, H), and Oil Red O (orange-red, I) staining confirm osteogenic, chondrogenic, and adipogenic differentiation, respectively. (scale bar = 100 μm). (**c**) Immunofluorescence detection (scale bar = 20 μm).

In vitro isolated and cultured Luopan Mountain pig pBMSCs formed visible colonies after 3 days of seeding. Cells exhibited spindle-shaped and vortex-like arrangements, with proliferation slowing as passage numbers increased (Figure 1a (A–F)). These cells showed multipotent differentiation potential: After 21 days of osteogenic induction, calcium nodules formed (alizarin red staining, Figure 1b (G)); after 17 days of chondrogenic induction, proteoglycan aggregation appeared (alizarin blue staining, Figure 1b (H)); and after 7 days of adipogenic induction, lipid droplets were observed (Oil Red O staining, Figure 1b (I)). Immunofluorescence results showed pBMSCs express CD29, CD44, CD73, CD90, and CD105, but not the hematopoietic stem cell marker CD34 (Figure 1c).

### 3.2. Liver-Targeted Homing and Improved Survival Rate of pBMSCs

**Figure 2:**
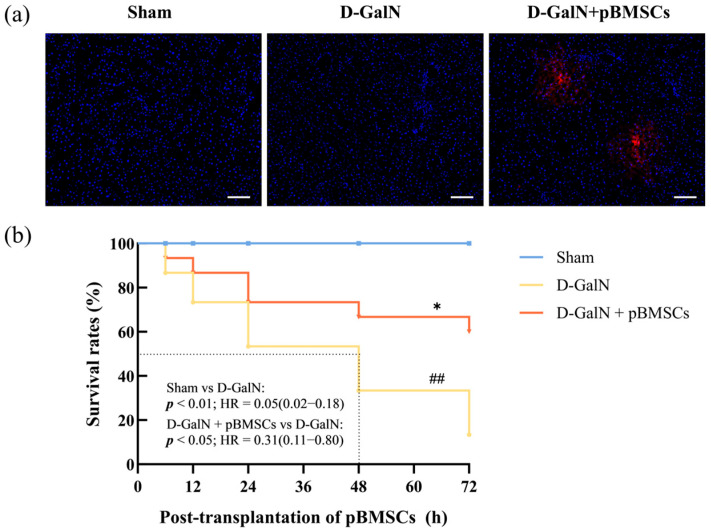
Liver-targeted homing and survival analysis of pBMSCs. (**a**) Hepatic distribution of CM-Dil-labeled pBMSCs (red) (scale bar = 100 μm). (**b**) Survival curve: pBMSC transplantation significantly improved survival rates in ALF rats (^##^ *p* < 0.01 vs. Sham; * *p* < 0.05 vs. D-GalN group; *n* = 15).

At 24 h after tail-vein injection of CM-Dil-labeled pBMSCs, fluorescence microscopy revealed their colonization within the injured liver (Figure 2a). The survival analysis results showed (Figure 2b) that the survival rate in the Sham group was 100%. Compared with the D-GalN group, pBMSC transplantation significantly improved rat survival rates (*p* < 0.05, HR = 0.31, 95% CI: 0.11–0.80), reducing the risk of death by 69%. This indicates that pBMSC transplantation exerts a significant protective effect against D-GalN-induced acute liver injury in rats.

### 3.3. pBMSC Transplantation Alleviates Histopathological Damage in the Liver Tissue of ALF Rats

**Figure 3:**
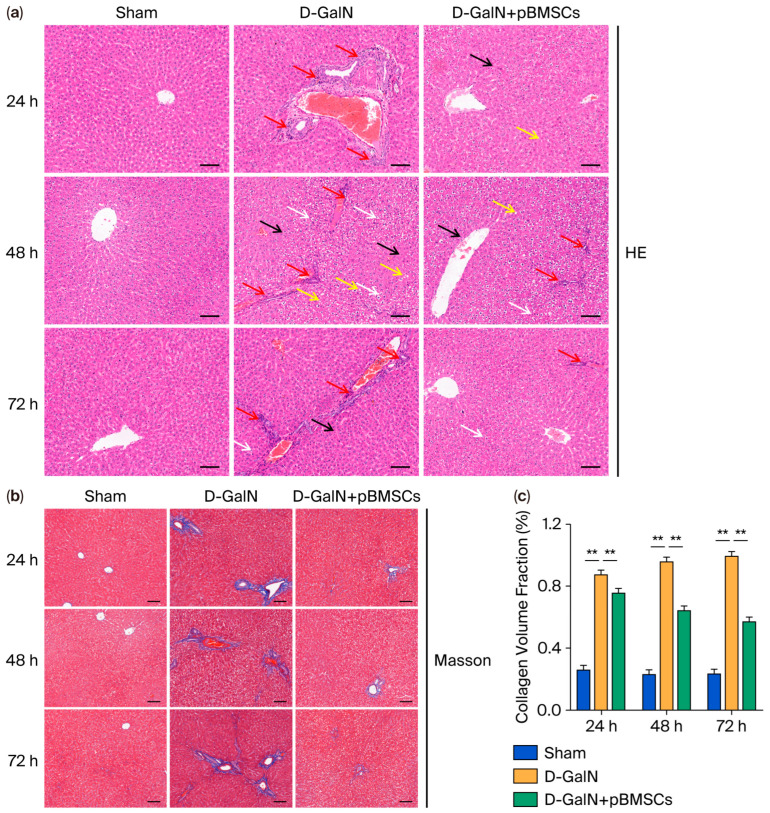
Effects of pBMSC transplantation on histopathological damage in ALF rat liver tissue. (**a**) Histopathological changes assessed with HE staining. Red arrows: Inflammatory infiltration in portal areas; Yellow arrows: Hepatocyte steatosis; Black arrows: Focal necrosis; White arrows: Hepatocyte edema (scale bar = 100 μm). (**b**) Masson’s trichrome staining for collagen deposition assessment (scale bar = 100 μm). (**c**) Quantitative analysis of collagen area (mean ± SD; ** *p* < 0.01).

Hepatic tissue HE staining revealed (Figure 3a) that the D-GalN group exhibited significantly worsened hepatocyte edema, steatosis, and focal necrosis compared with the Sham group along with marked inflammatory infiltration in the portal areas; these lesions were markedly reduced in the pBMSC transplantation group. Masson staining (Figure 3b,c) revealed extensive collagen deposition (blue staining) in the portal areas and perisinusoidal regions of the D-GalN group, whereas the pBMSC transplantation group exhibited markedly reduced collagen fiber deposition.

### 3.4. pBMSC Transplantation Improves Liver Function and Metabolic Marker Levels in ALF Rats

**Figure 4:**
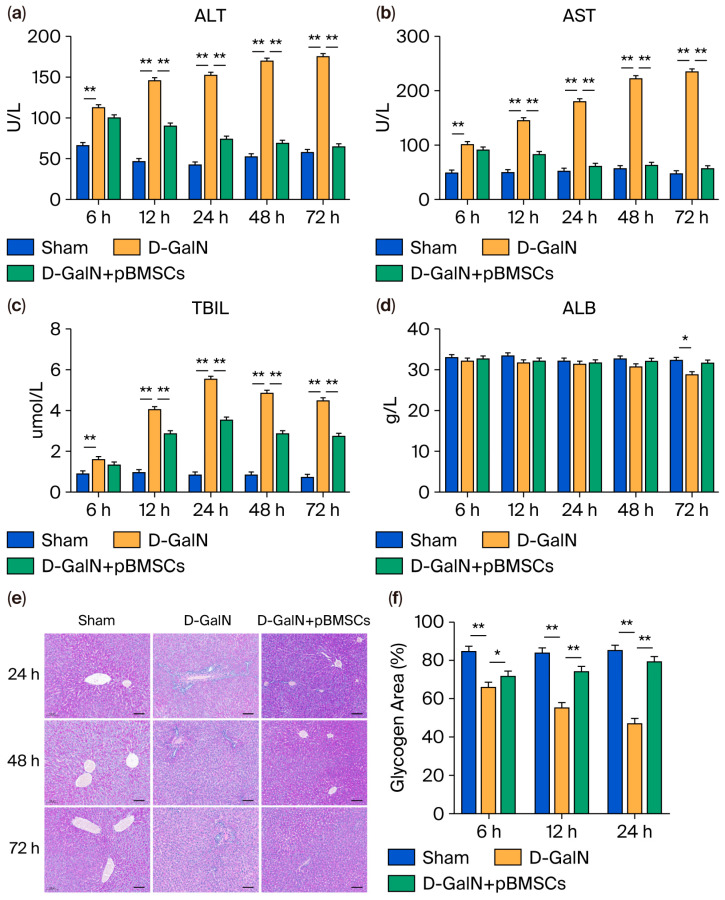
Effects of pBMSC transplantation on liver function and hepatic glycogen in ALF rats. ELISA detection of serum ALT (**a**), AST (**b**), TBIL (**c**), and ALB (**d**) levels. (**e**) PAS staining showing glycogen distribution (scale bar = 100 μm). (**f**) Quantitative analysis of glycogen area (mean ± SD; * *p* < 0.05, ** *p* < 0.01).

The ELISA analysis results demonstrated that, relative to the Sham group, the D-GalN group exhibited serum ALT, AST, and TBIL levels were significantly increased (*p* < 0.01; Figure 4a–c); upon pBMSC transplantation, the levels of all these three serum markers decreased significantly (*p* < 0.05), whereas the serum ALB level in the D-GalN group showed a reduction that was dependent on time (Figure 4d). Although ALB showed an upward trend after pBMSC transplantation, no significant difference was found (*p* > 0.05). PAS staining revealed (Figure 4e,f) reduced glycogen granules and weakened staining in the D-GalN group, whereas glycogen content and staining intensity were markedly higher in the pBMSC transplantation group compared to the D-GalN group, suggesting that pBMSC transplantation effectively promotes glycogen synthesis and improves metabolism.

### 3.5. pBMSC Transplantation Inhibits Proinflammatory Cytokine Release

**Figure 5:**
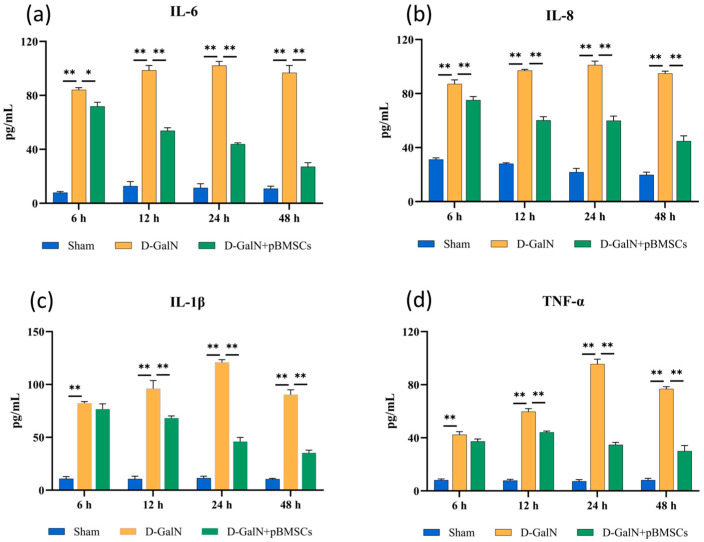
Effects of pBMSC transplantation on serum inflammatory factors in ALF rats. ELISA detection of IL-6 (**a**), IL-8 (**b**), IL-1β (**c**), and TNF-α (**d**) levels (mean ± SD; * *p* < 0.05, ** *p* < 0.01; *n* = 3).

Serological analysis revealed (Figure 5a–d) that serum levels of the proinflammatory cytokines IL-6, IL-8, IL-1β, and TNF-α were significantly elevated in the D-GalN group compared to the Sham group (*p* < 0.01). These levels continued to rise between 6 and 24 h post-treatment before declining after 48 h. The concentrations of all factors in the pBMSC transplantation group were significantly lower than those in the D-GalN group (*p* < 0.01).

### 3.6. pBMSC Transplantation Inhibits Apoptosis of Liver Cells in ALF Rats

**Figure 6:**
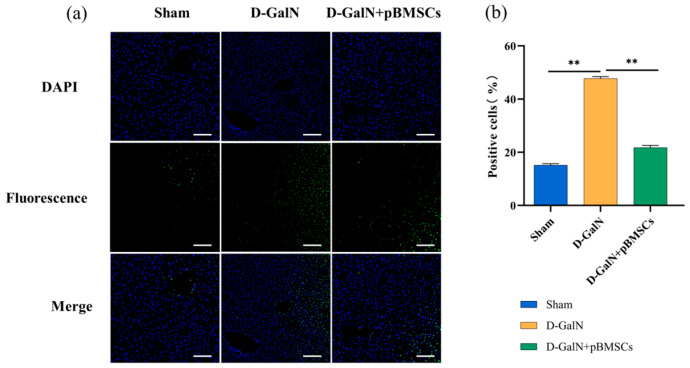
Effects of pBMSC transplantation on hepatocyte apoptosis in ALF rats. (**a**) TUNEL staining (scale bar = 100 μm). (**b**) Quantitative analysis of positive cells (mean ± SD; ** *p* < 0.01; *n* = 3).

TUNEL assays revealed (Figure 6a,b) that the number of apoptotic cells in the D-GalN group had was significantly higher than in the Sham group (*p* < 0.01) at 24 h after pBMSC transplantation, while the pBMSC transplantation group exhibited a significant reduction compared to the D-GalN group (*p* < 0.01).

### 3.7. Regulation of Liver Cell Proliferation in ALF Rats by pBMSC Transplantation

**Figure 7:**
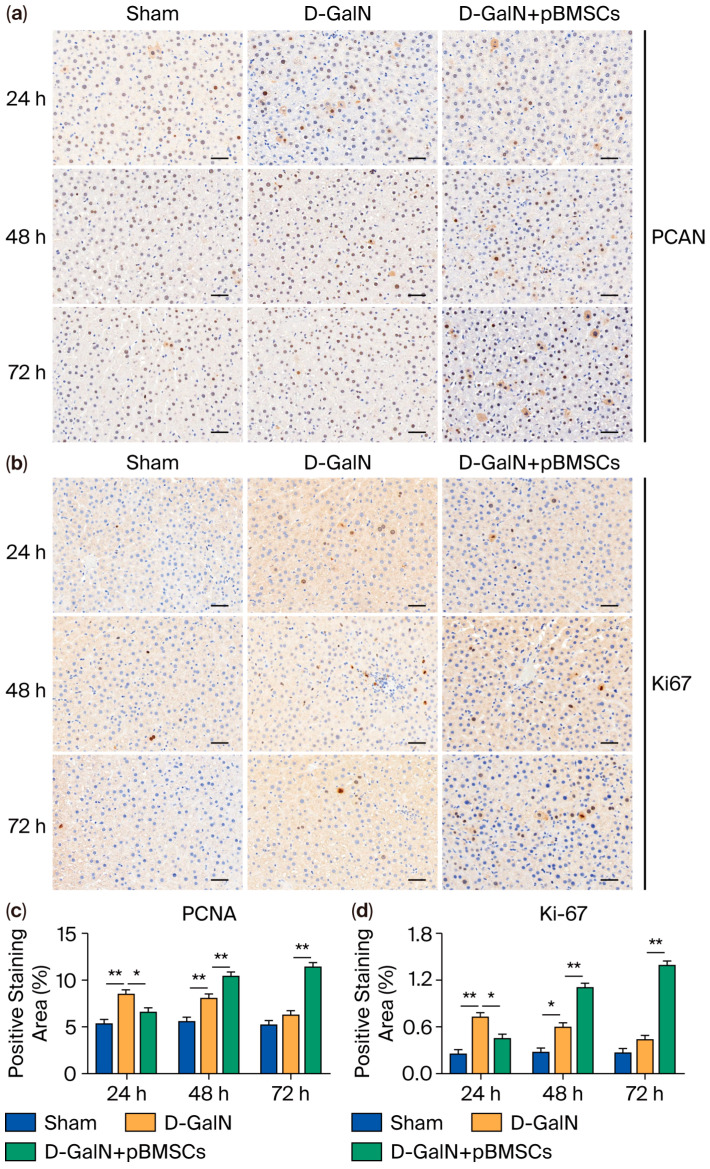
Effects of pBMSC transplantation on hepatocyte proliferation in ALF rats. (**a**) PCNA and (**b**) Ki-67 immunohistochemical staining. Quantitative analysis of PCNA (**c**) and Ki-67 (**d**) positive cells. (mean ± SD; * *p* < 0.05, ** *p* < 0.01; *n* = 3).

Ki-67 and PCNA immunohistochemical staining revealed the following (Figure 7a–c): At 24 h, the D-GalN group had a significantly larger proliferation-positive area in comparison to the Sham group (*p* < 0.01). At 48 h and 72 h, the pBMSC transplantation group exhibited a significantly higher proliferation-positive area than the D-GalN group (*p* < 0.01), while the proliferation-positive area of the D-GalN group decreased in a manner that was related to the passage of time.

### 3.8. pBMSC Transplantation Upregulates Serum Growth Factor Levels in ALF Rats

**Figure 8:**
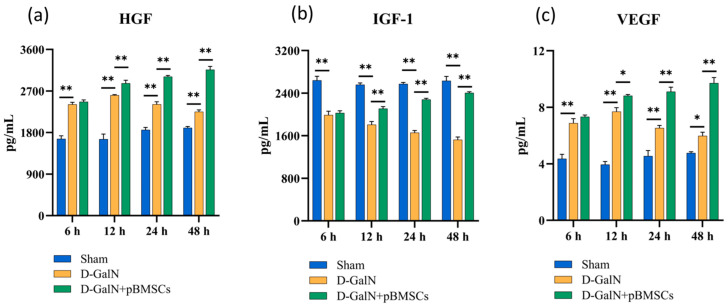
Effects of pBMSC transplantation on serum growth factor levels in ALF rats. ELISA detection of HGF (**a**), IGF-1 (**b**), and VEGF (**c**) expression (mean ± SD; * *p* < 0.05, ** *p* < 0.01; *n* = 3).

ELISA analysis revealed that serum IGF-1 levels in D-GalN-induced ALF rats showed a sustained significant decrease from 6 to 48 h (*p* < 0.01; Figure 8b) compared with the Sham group, while HGF (Figure 8a) and VEGF (Figure 8c) levels initially increased and then decreased. pBMSC transplantation significantly elevated all growth factor levels (*p* < 0.01), indicating that pBMSC transplantation effectively upregulates serum growth factor expression in ALF rats.

### 3.9. Regulation of Inflammatory, Fibrotic, and Apoptotic Gene Expression in Liver Tissue of ALF Rats Following pBMSC Transplantation

**Figure 9:**
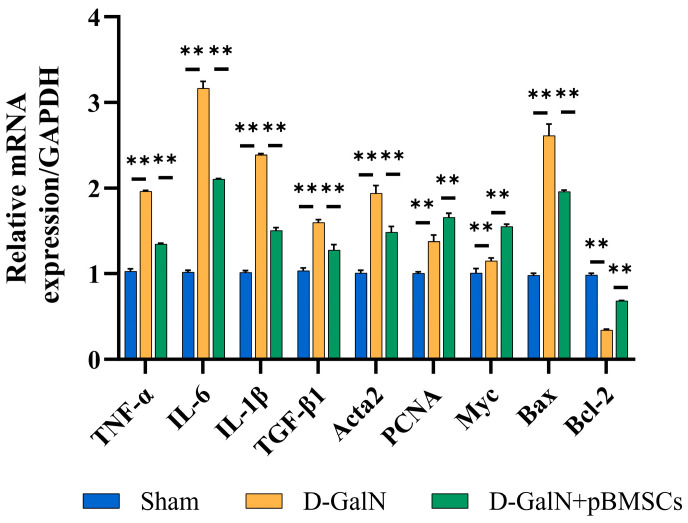
Effects of pBMSC transplantation on gene expression in liver tissue of ALF rats. qPCR detection of mRNA levels for genes related to inflammation, fibrosis, apoptosis, and proliferation (mean ± SD; ** *p* < 0.01).

The qPCR results (Figure 9) revealed that the D-GalN group exhibited significantly increased mRNA expression of proinflammatory factors (*TNF-α*, *IL-6*, *IL-1β*) and pro-fibrotic factors (*TGF-β1* and *Acta2*) compared with the Sham group. The proapoptotic gene *Bax* was significantly upregulated (*p* < 0.01), while the expression of the antiapoptotic gene *Bcl-2* was significantly downregulated (*p* < 0.01). Following pBMSC transplantation, the expression of the aforementioned genes was effectively reversed, and mRNA expression levels of proliferation-related genes (*PCNA* and *Myc*) were significantly enhanced (*p* < 0.01). These findings indicate that pBMSCs can synergistically suppress inflammation, fibrosis, and apoptosis at the transcriptional level while promoting proliferation, providing a molecular basis for improving liver injury.

### 3.10. pBMSC Transplantation Alleviates Liver Damage by Activating the PI3K/Akt/mTOR Pathway and Modulating the Expression of Proteins Associated with Apoptosis

**Figure 10:**
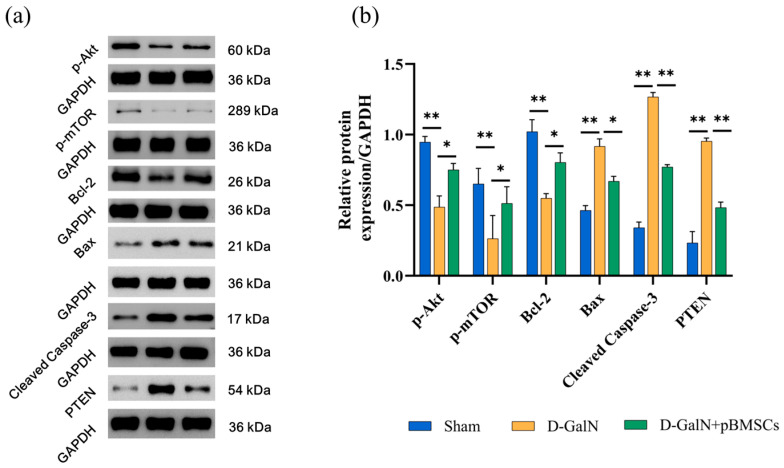
Effects of pBMSC transplantation on the PI3K/Akt/mTOR pathway and apoptosis-related proteins in ALF rats. (**a**) Western blot analysis results. (**b**) Quantitative analysis of p-Akt, p-mTOR, Bcl-2, Bax, Cleaved Caspase-3, and PTEN (mean ± SD; * *p* < 0.05, ** *p* < 0.01; *n* = 3).

Western blot results (Figure 10a,b and Figure S1) demonstrated that, in contrast to the Sham group, the D-GalN group had notably downregulated expression of p-Akt (*p* < 0.01), p-mTOR (*p* < 0.01); meanwhile, the anti-apoptotic protein Bcl-2 decreased significantly (*p* < 0.01), while the pro-apoptotic factors Bax, Cleaved Caspase-3, and PTEN were all significantly upregulated (all *p* < 0.01); after pBMSC transplantation, the phosphorylation levels of p-Akt (*p* < 0.05) and p-mTOR (*p* < 0.05) increased remarkably, the expression of PTEN (*p* < 0.01) decreased significantly, and the regulation of apoptosis was improved at the same time, evidenced by upregulated Bcl-2 (*p* < 0.05) and downregulated Bax (*p* < 0.05) and Cleaved Caspase-3 (*p* < 0.01),which indicates that pBMSCs may alleviate liver injury by regulating inhibit apoptosis by activating the PI3K/Akt/mTOR pathway and inhibiting PTEN.

## 4. Discussion

This study provides the first evidence that pBMSCs derived from Luopan Mountain pigs exert hepatoprotective and regenerative effects in a D-GalN-induced ALF rat model. The experimental results demonstrate that tail-vein transplantation of pBMSCs significantly improves survival rates in ALF rats, mitigates hepatic histopathological damage, and enhances key liver function indicators (ALT, AST, and TBIL) alongside liver glycogen metabolism levels. At the mechanistic level, we revealed that pBMSCs drive liver function recovery and regeneration by synergistically activating the PI3K/Akt/mTOR pro-survival signaling pathway and downregulating the key negative regulator PTEN. These findings not only provide a novel pBMSC-based therapeutic strategy for ALF, but also lay a theoretical foundation for the clinical translation of xenogeneic stem cell transplantation.

ALB serves as a key indicator for assessing hepatic synthetic and metabolic functions, with its levels reflecting the extent of hepatocyte damage and the state of regenerative repair [27]. He et al. [28] demonstrated in a lipopolysaccharide (LPS)-induced acute-to-chronic liver injury (ACLI) rat model that following hUC-MSC intervention, serum ALB levels in rats steadily increased, returning to near-normal ranges by week 6. Although an upward trend in ALB levels in the pBMSC-treated group was observed in this study, the intergroup difference was not statistically significant (*p* > 0.05). This phenomenon may be related to the relatively long half-life of ALB [29], and the serum concentration changes which lag behind those of acute liver enzyme markers such as ALT and AST.

Ki-67 and PCNA are two classic markers for evaluating hepatocyte proliferation, and their positive areas directly reflect the proportion of proliferating hepatocytes. Ki-67 is a nuclear antigen that remains expressed throughout all active phases of the cell cycle, including G1, S, G2, and M [30,31]. PCNA, as an auxiliary protein of DNA polymerase, participates in DNA replication and repair processes [32,33]. Studies have indicated that, in rat liver resection models, Ki-67 and PCNA expression positively correlate with hepatocyte proliferation and liver regeneration [34]. This study found that Ki-67 and PCNA expression in ALF rats gradually decreased at 48–72 h, suggesting that sustained injury leads to suppressed proliferative activity. By contrast, the pBMSC transplantation group showed significantly higher expression than the D-GalN group, suggesting pBMSCs can boost proliferation and regeneration of damaged hepatocytes, consistent with Wang et al.’s findings [35]. This study found that pBMSC transplantation reduced serum levels of proinflammatory factors (IL-6, IL-8, IL-1β, TNF-α) in D-GalN-induced ALF rats, while upregulating the expression of IGF-1, HGF, and VEGF. These growth factors may synergistically activate the PI3K/Akt/mTOR pathway: HGF mediates pathway activation via the c-Met receptor [36], while IGF-1 does so through IGF-1R, thereby regulating cell proliferation, migration, and metabolism [37]. VEGF improves hepatic microcirculation by promoting angiogenesis [38]. The combined effects of pathway activation and proinflammatory factor downregulation may synergistically mitigate liver injury and promote repair through multiple mechanisms, including promoting macrophage polarization toward the M2 phenotype, suppressing excessive T-cell responses, and supporting hepatocyte regeneration [39].

This study revealed the key molecular mechanisms underlying the hepatoprotective effects mediated by pBMSCs through Western blot analysis. Results showed pBMSC transplantation significantly increased p-Akt and p-mTOR phosphorylation in ALF rat liver tissues, indicating activation of the PI3K/Akt/mTOR signaling axis. This pathway serves as a core mechanism driving hepatocyte survival, proliferation, and metabolic adaptation [40,41]. Notably, PTEN expression was significantly downregulated in the pBMSC-transplanted group, suggesting that pBMSCs not only activate positive signaling pathways but also enhance signal transduction efficiency by suppressing key negative regulators [42]. This finding aligns with the study by Jung et al. [43], who demonstrated that activation of the farnesoid X receptor (FXR) in zebrafish models enhances PTEN activity to inhibit the PI3K/Akt/mTOR pathway, thereby impeding liver progenitor cell (LPC)-mediated regeneration. This provides reverse validation of PTEN’s negative regulatory role. Existing research indicates [44] that PTEN loss or dysfunction can lead to excessive activation of Akt, thereby enhancing the activity of mTORC1 which, in turn, influences cell growth, proliferation, and metabolism by regulating downstream effector molecules such as S6K and 4E-BP1 [45,46].

PI3K/Akt/mTOR pathway activation also modulates the expression of apoptosis-related proteins [47]. This study found that pBMSC transplantation significantly upregulated the expression of the anti-apoptotic protein Bcl-2 in the liver tissue of ALF rats while simultaneously downregulating the expression of pro-apoptotic factors Bax and Cleaved Caspase-3, suggesting that it may reduce apoptosis execution by blocking the mitochondrial apoptosis pathway [48]. The Bcl-2/Bax ratio—a key indicator for assessing apoptotic propensity [49]—has been validated in liver fibrosis models, where tannic acid A inhibits hepatocyte apoptosis by elevating this ratio [50]. Similarly, Chen et al. [51] demonstrated that MYBL2 overexpression promotes tumor cell survival in gastric cancer by modulating the Bcl-2/Bax ratio.

However, this study still has limitations. Although the rat model yielded positive results, rats lack pre-existing anti-pig antibodies (such as anti-Gal) that are present in humans, making it impossible to simulate human xenogeneic immune responses (where these antibodies recognize the α-Gal epitope on pig cells, triggering strong rejection) [52,53,54]. Pig-to-human transplantation also faces obstacles such as complement activation and cellular immunity [55,56]. Future efforts should leverage gene editing technologies and utilize humanized mouse or non-human primate models for further evaluation to advance clinical translation [57,58].

## 5. Conclusions

In summary, this study is the first to demonstrate that transplanted pBMSCs from Luopan Mountain pigs reduce PTEN expression and activate the PI3K/Akt/mTOR pathway. The altered expression of molecules within this pathway correlates strongly with improved liver function, suppressed hepatocyte apoptosis, and enhanced proliferation. These findings suggest that the PTEN-PI3K/Akt/mTOR axis may represent a key regulatory pathway mediating liver regeneration by pBMSCs. Following pBMSC transplantation, serum levels of HGF, IGF-1, and VEGF were upregulated, thereby enhancing hepatocyte proliferation capacity. Serum ALT, AST, and TBIL levels decreased, and impaired hepatic glycogen synthesis was improved. Concurrently, the mitochondrial apoptosis pathway was blocked via regulation of the expression of Bcl-2, Bax, and Cleaved Caspase-3. This study not only provides a novel xenogeneic stem cell-based therapeutic strategy for ALF, but also establishes a scientific foundation for the safe clinical application of porcine-derived stem cells in liver disease.

## Data Availability

The original data supporting the conclusions of this paper are available upon request from the corresponding author.

## Supplementary Materials

The following supporting information can be downloaded at: https://www.mdpi.com/article/10.3390/biology14101363/s1, Figure S1: Original Western Blot; Figure S2: Melting Curve (for qPCR); Table S1: Primers for quantitative real-time PCR analysis.

## Abbreviations

The following abbreviations are used in this manuscript:

ALF	Acute Liver Failure
pBMSCs	Porcine Bone Marrow Mesenchymal Stem Cells
D-GalN	D-galactosamine
