# Supplementary material for: Luopan Mountain Pig Bone Marrow Mesenchymal Stem Cells Promote Liver Regeneration in D-Galactosamine-Induced Acute Liver Failure Rats by Regulating the PTEN-PI3K/Akt/mTOR Pathway
Source: Biology (Basel). 2025 Oct 5;14(10):1363. doi: 10.3390/biology14101363 (PMC12562111; doi:10.3390/biology14101363)
Supplement: Supplementary file 1 [file biology-14-01363-s001.zip › Table S1.pdf]

Table S1. Primers for quantitative real-time PCR analysis.

Gene	Primer
GAPDH	ACAGCAACAGGGTGGTGGAC<br>TTTGAGGGTGCAGCGAACTT
TNF-α	CAGACCCTCACACTCAGATCAT<br>AGATAAGGTACAGCCCATCTGC
IL-6	CTCTCCGCAAGAGACTTCCA<br>TCTCCTCTCCGGACTTGTGAA
IL-1β	CCTGTGTGATGAAAGACGGC<br>TATGTCCCGACCATTGCTGT
TGF-β1	CCCTACATTTGGAGCCTGGA<br>CGCACGATCATGTTGGACAA
Acta2	GGATCAGCGCCTTCAGTTCT<br>CAGGGCTAGAAGGGTAGCAC
PCNA	ATCTAGACGTCGCAACTCCG<br>GCTGCACTAAGGAGACGTGA
BAX	AAGAAGCTGAGCGAGTGTCT<br>CCAGTTGAAGTTGCCGTCTG
Bcl-2	GCCTTCTTTGAGTTCGGTGG<br>CTGAGCAGCGTCTTCAGAGA
Myc	CTCGGTGCAGCCCTATTTCA<br>TAGCGACCGCAACATAGGAC
