# Supplementary figures and images for: Luopan Mountain Pig Bone Marrow Mesenchymal Stem Cells Promote Liver Regeneration in D-Galactosamine-Induced Acute Liver Failure Rats by Regulating the PTEN-PI3K/Akt/mTOR Pathway
Source: Biology (Basel). 2025 Oct 5;14(10):1363. doi: 10.3390/biology14101363 (PMC12562111; doi:10.3390/biology14101363)

Figure S1 Original Wwstern Blot  
(1)

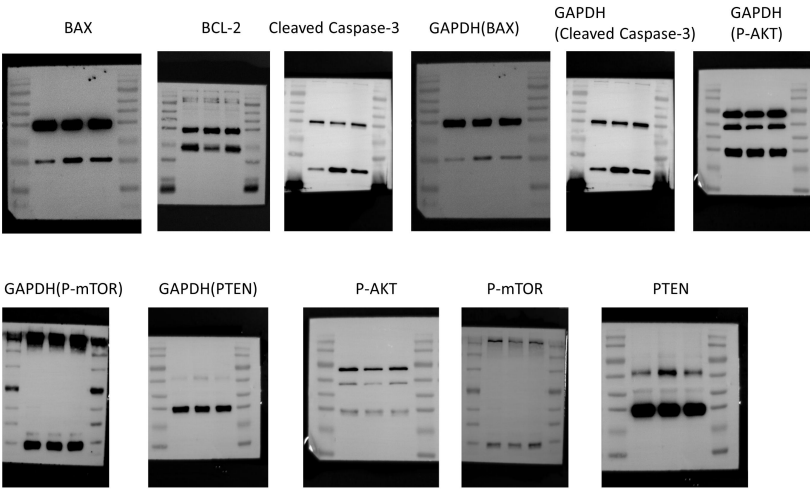

(2)

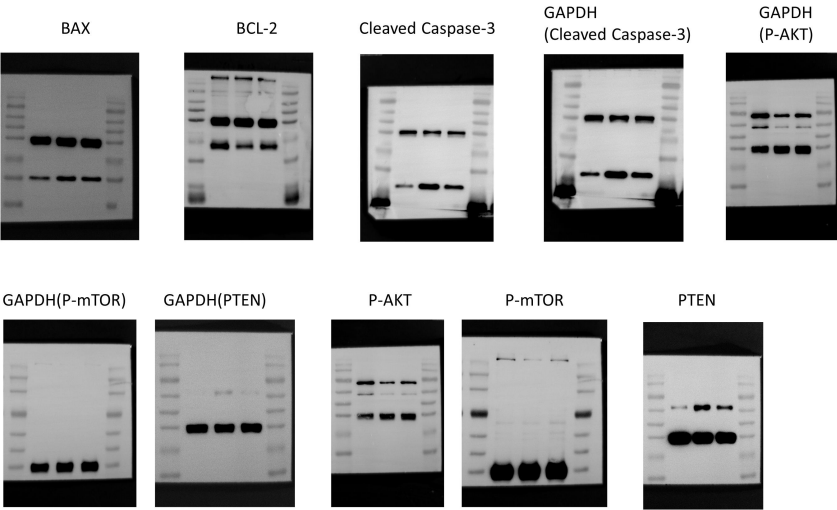

(3)

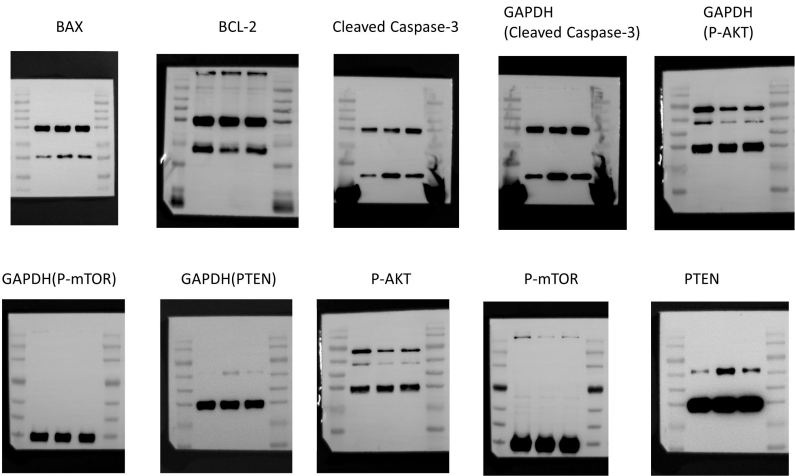

Supplement: Supplementary file 1 [file biology-14-01363-s001.zip › Figure S1.pdf]

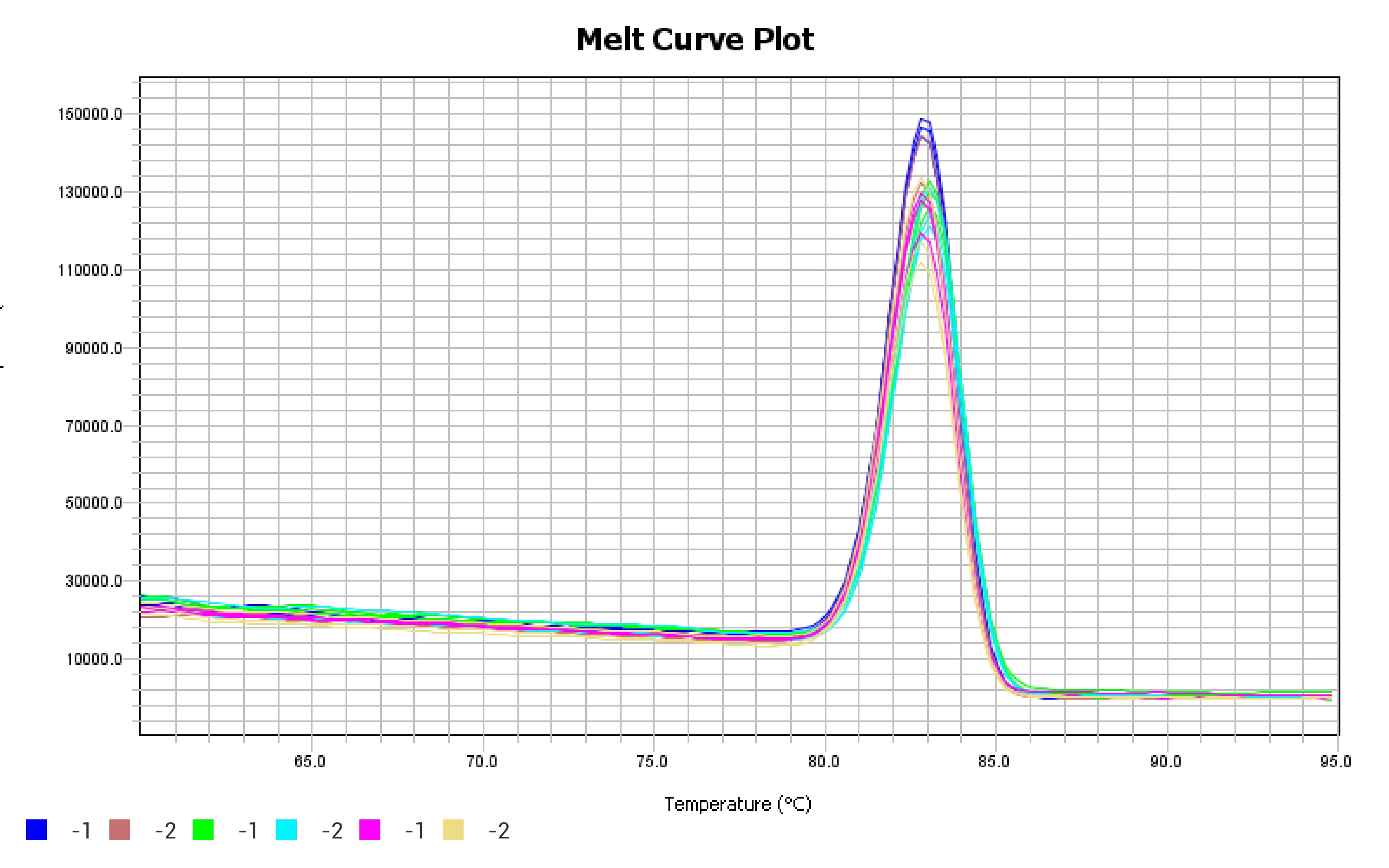

Supplement: Supplementary file 1 [file biology-14-01363-s001.zip › Figure S2-Melting Curve (for qPCR)/Melt Curve Plot-Rat1.2 Acta2.jpg]

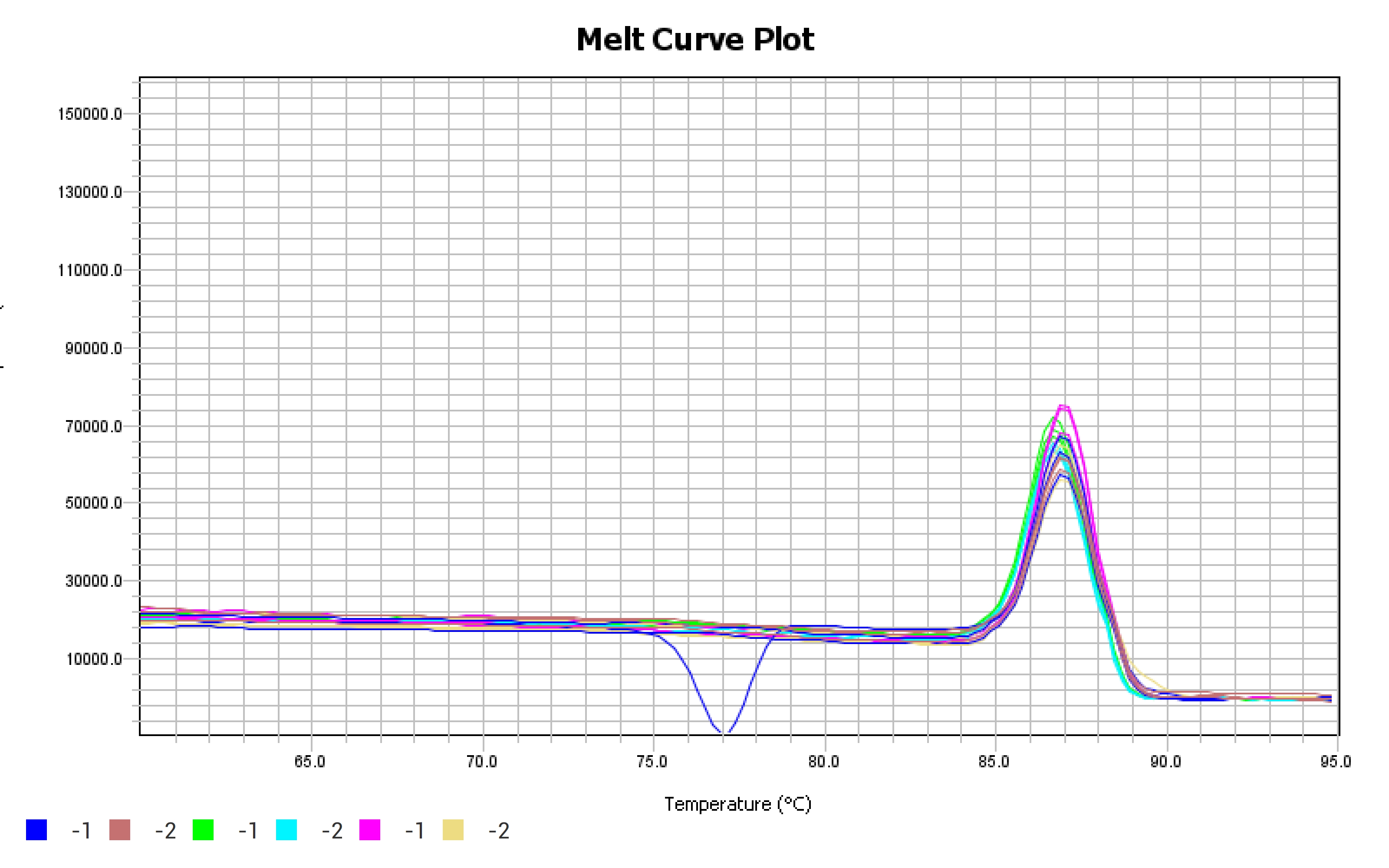

Supplement: Supplementary file 1 [file biology-14-01363-s001.zip › Figure S2-Melting Curve (for qPCR)/Melt Curve Plot-Rat1.2 BAX.jpg]

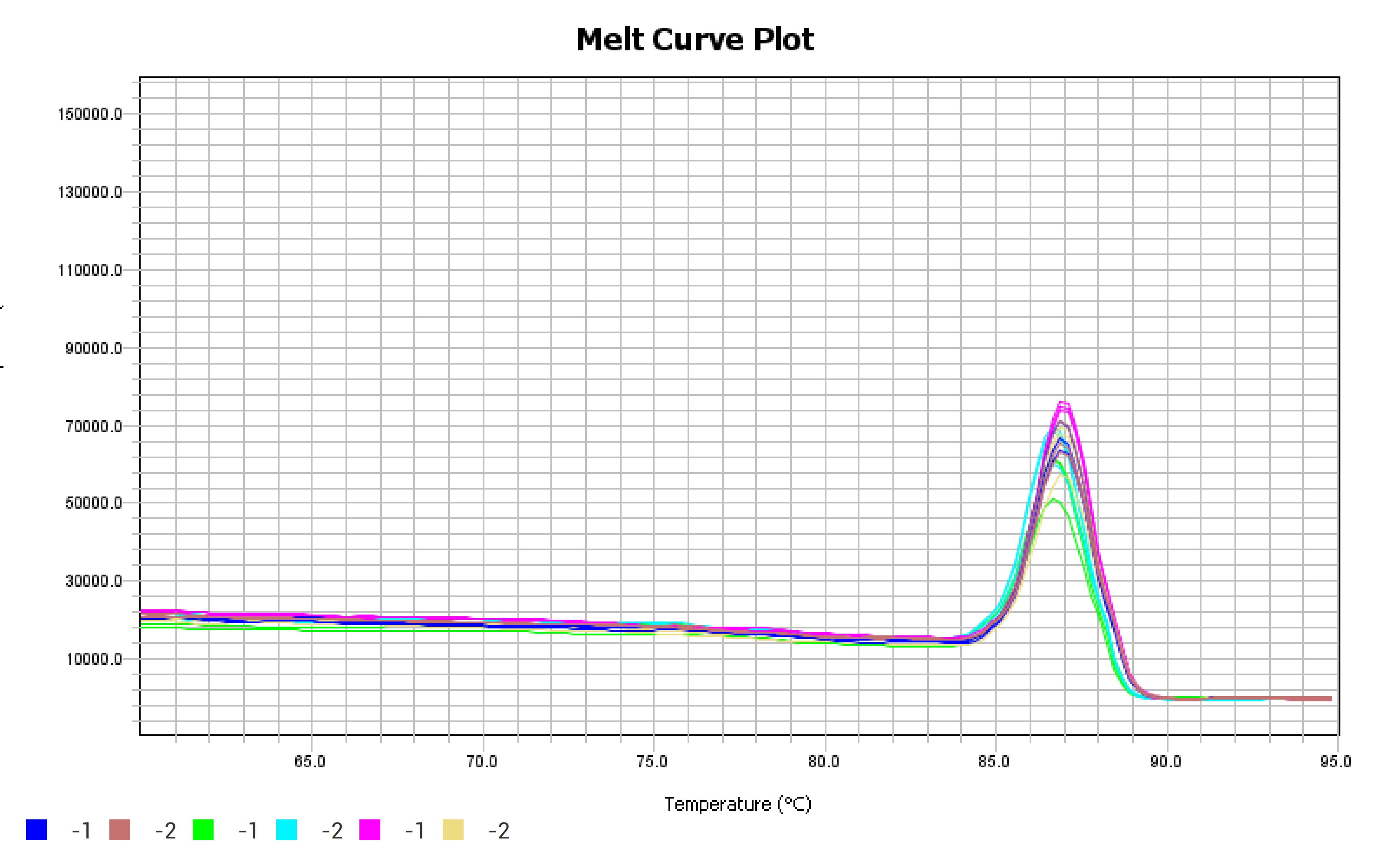

Supplement: Supplementary file 1 [file biology-14-01363-s001.zip › Figure S2-Melting Curve (for qPCR)/Melt Curve Plot-Rat1.2 Bcl-2.jpg]

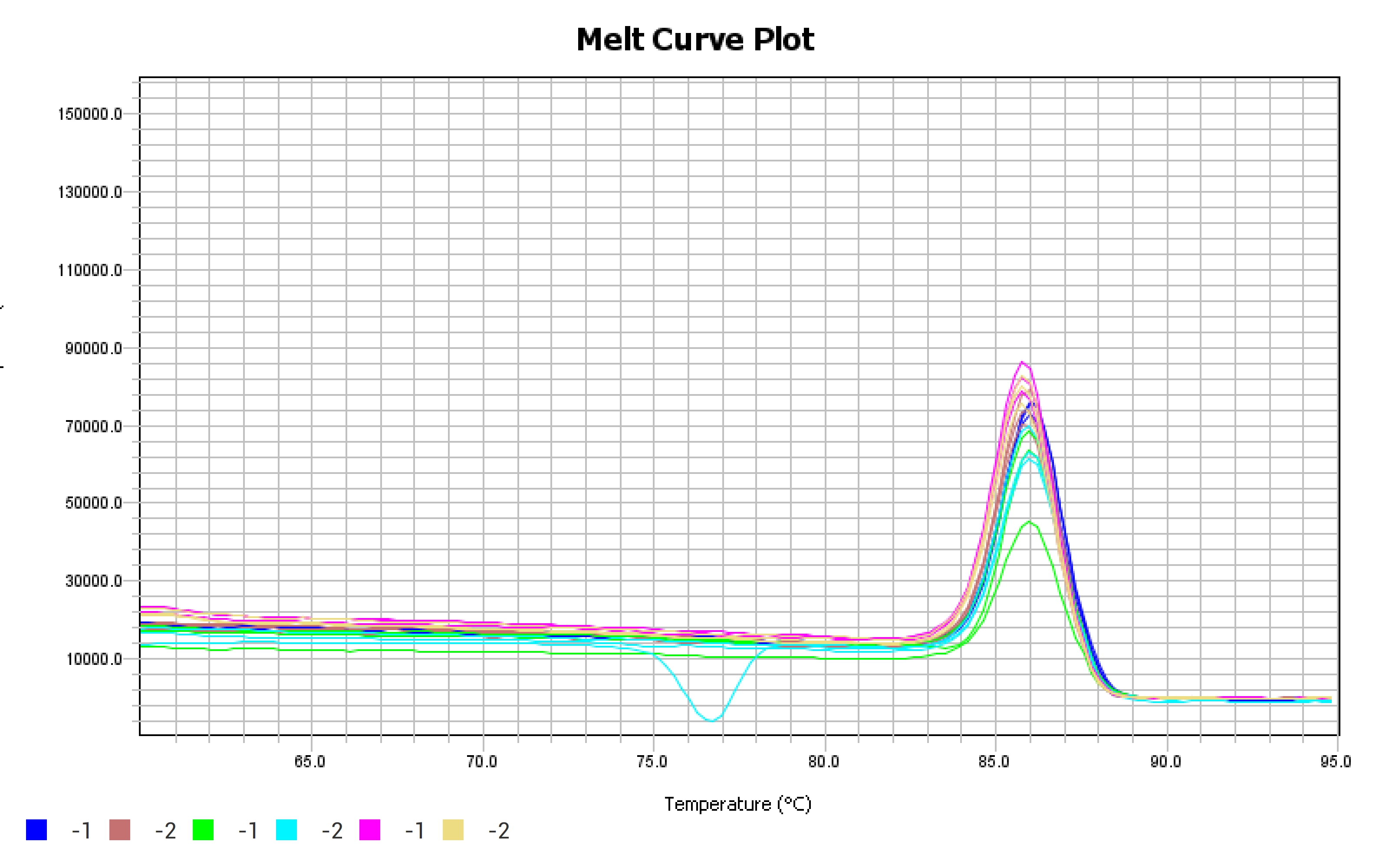

Supplement: Supplementary file 1 [file biology-14-01363-s001.zip › Figure S2-Melting Curve (for qPCR)/Melt Curve Plot-Rat1.2 GAPDH.jpg]

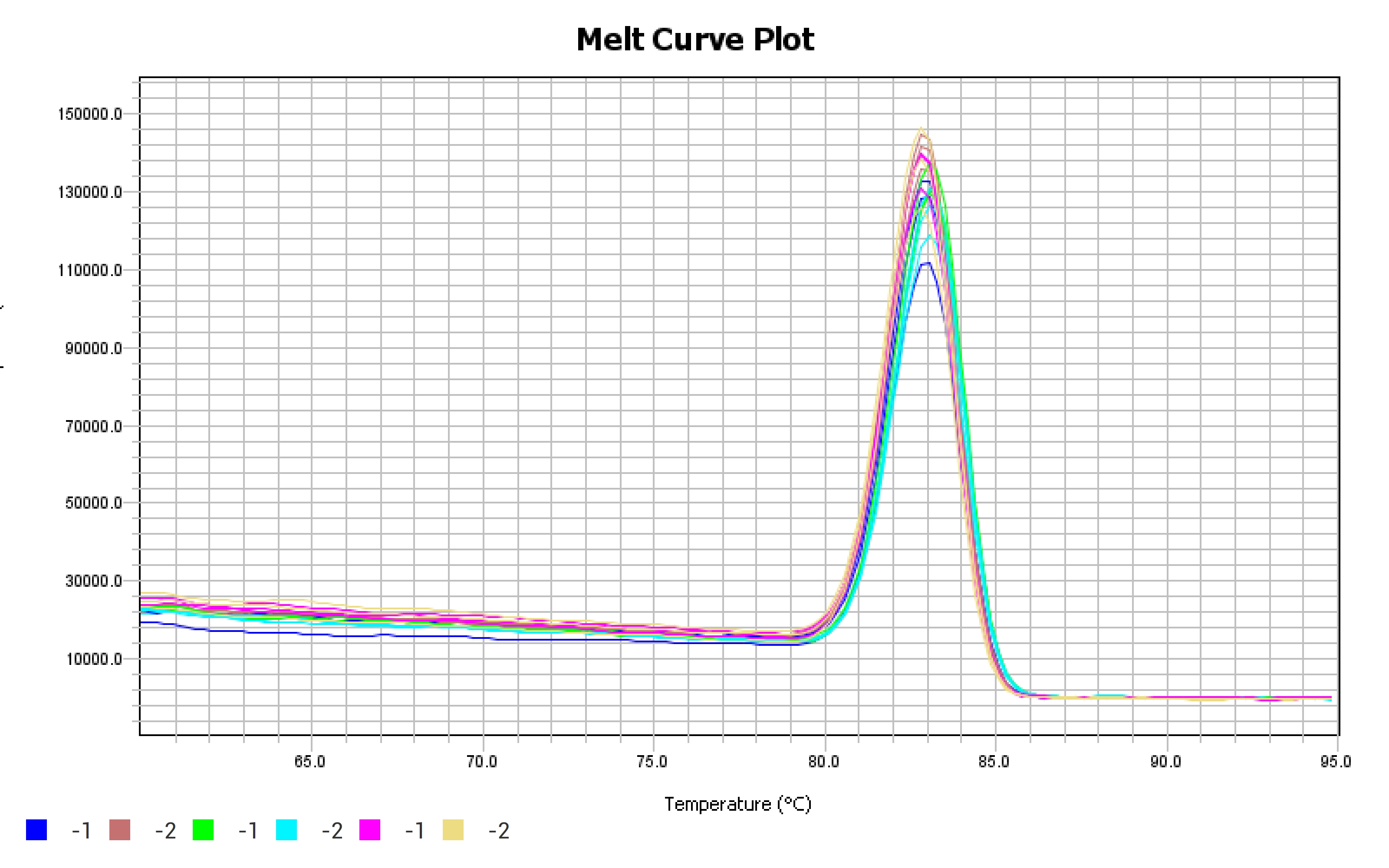

Supplement: Supplementary file 1 [file biology-14-01363-s001.zip › Figure S2-Melting Curve (for qPCR)/Melt Curve Plot-Rat1.2 IL-1a┬.jpg]

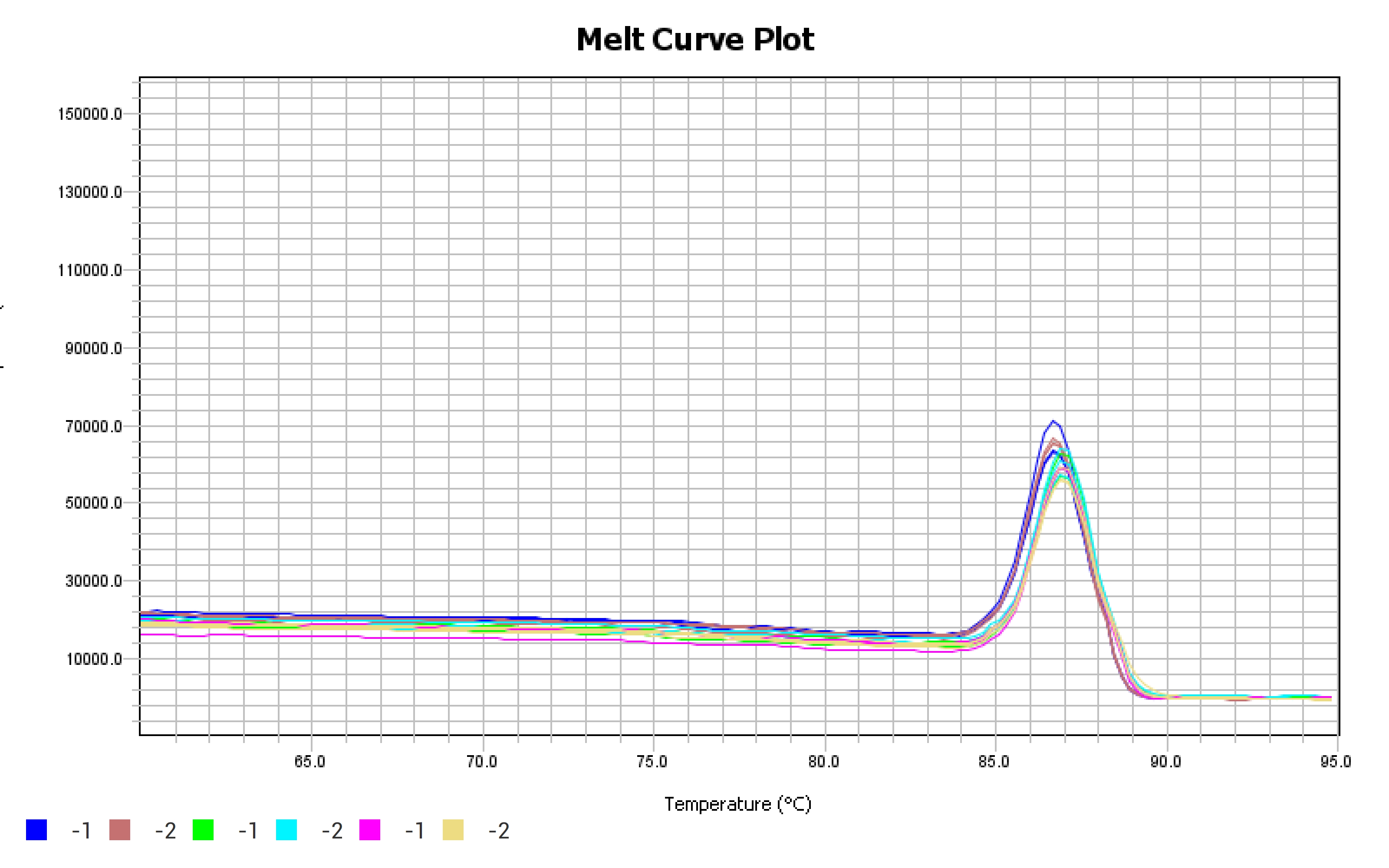

Supplement: Supplementary file 1 [file biology-14-01363-s001.zip › Figure S2-Melting Curve (for qPCR)/Melt Curve Plot-Rat1.2 IL-6.jpg]

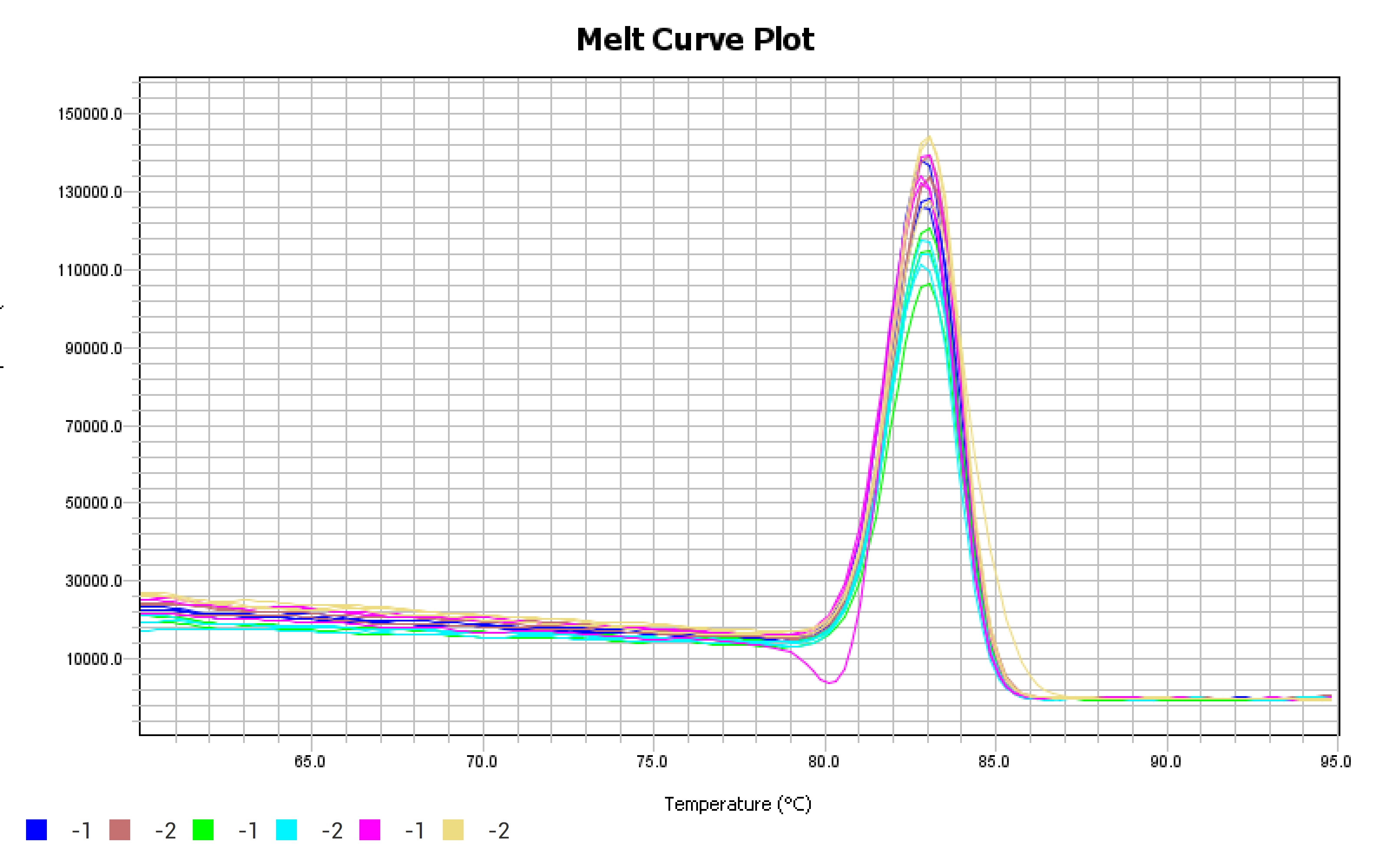

Supplement: Supplementary file 1 [file biology-14-01363-s001.zip › Figure S2-Melting Curve (for qPCR)/Melt Curve Plot-Rat1.2 Myc.jpg]

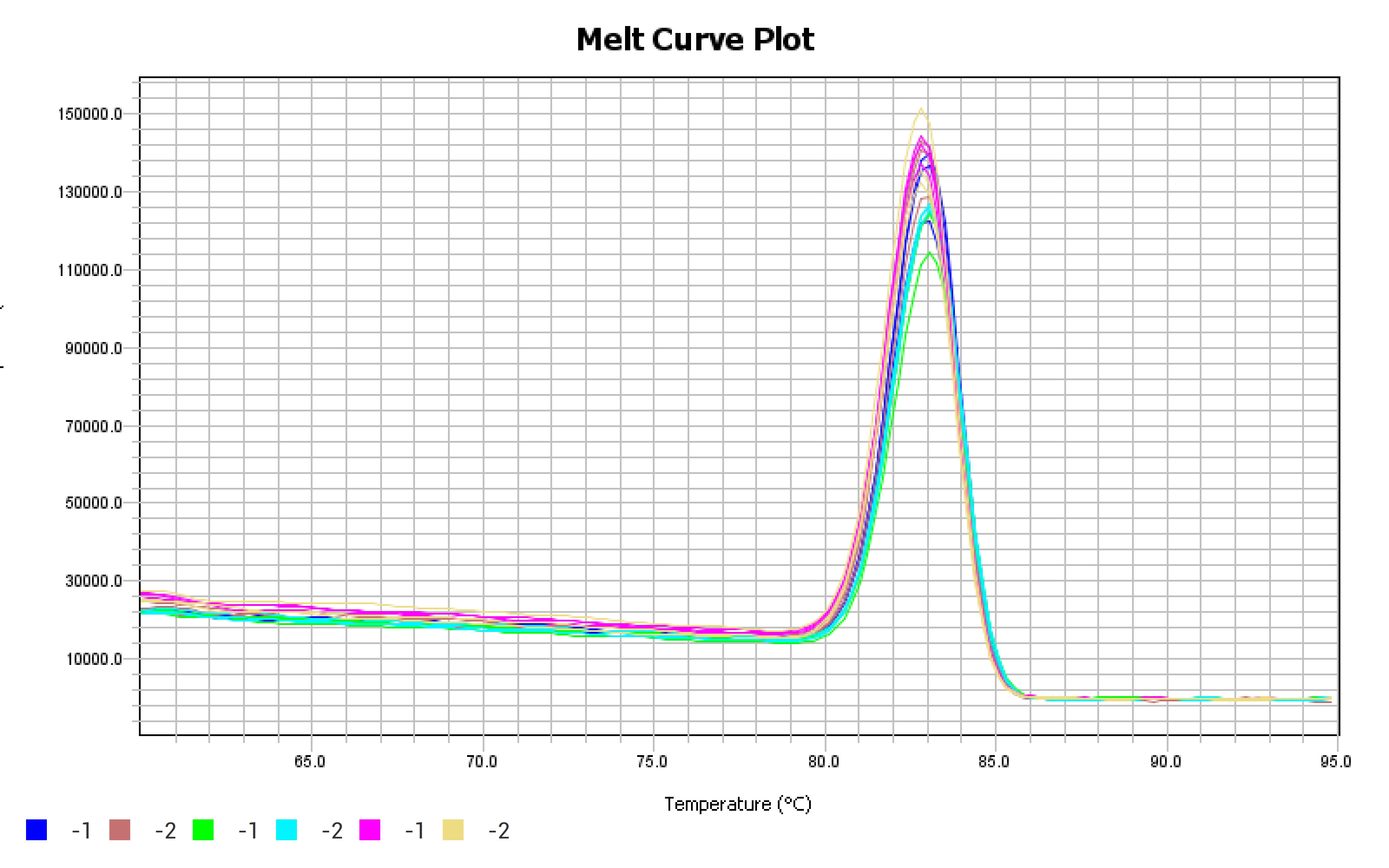

Supplement: Supplementary file 1 [file biology-14-01363-s001.zip › Figure S2-Melting Curve (for qPCR)/Melt Curve Plot-Rat1.2 PCNA.jpg]

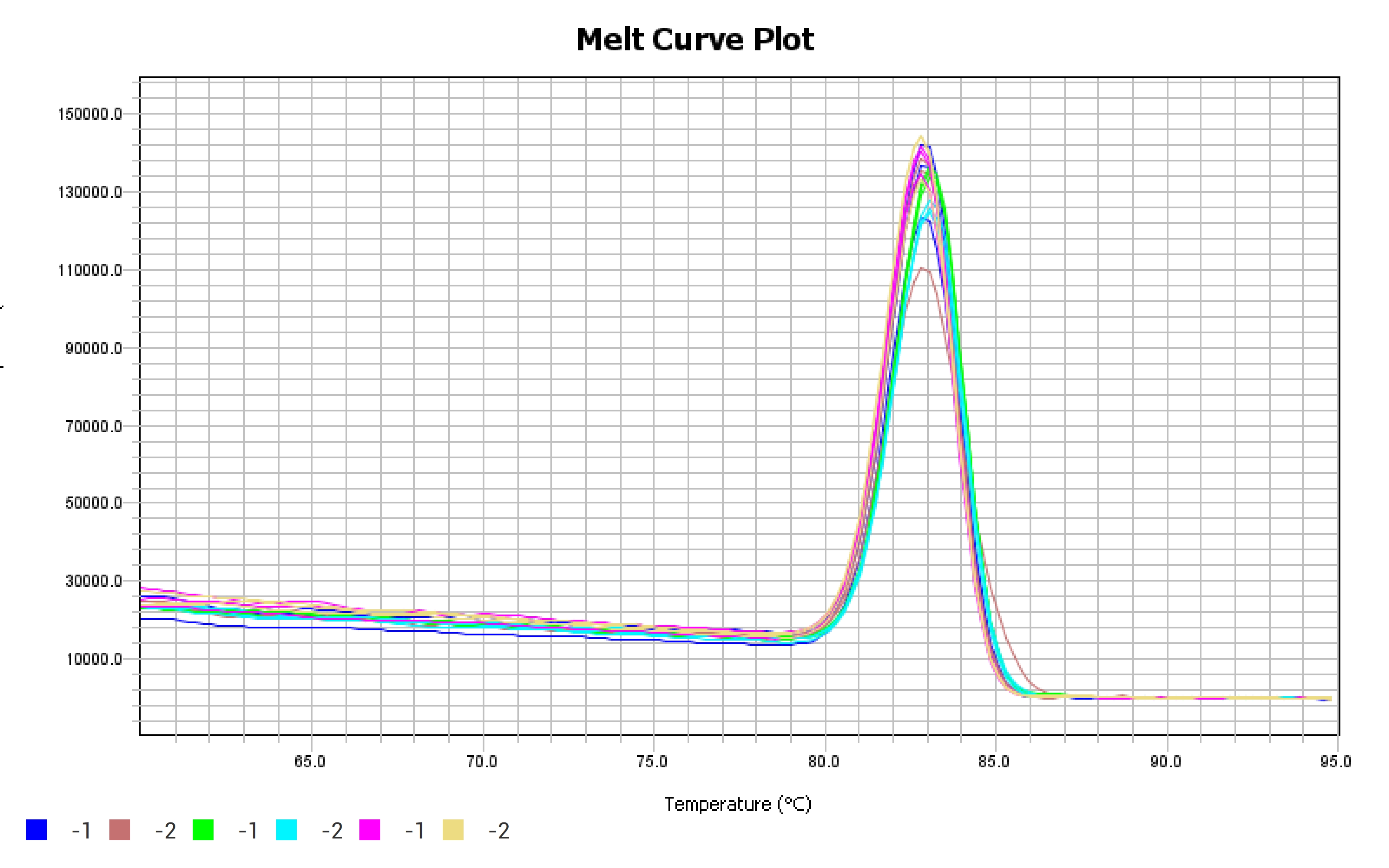

Supplement: Supplementary file 1 [file biology-14-01363-s001.zip › Figure S2-Melting Curve (for qPCR)/Melt Curve Plot-Rat1.2 TGF-a┬1.jpg]

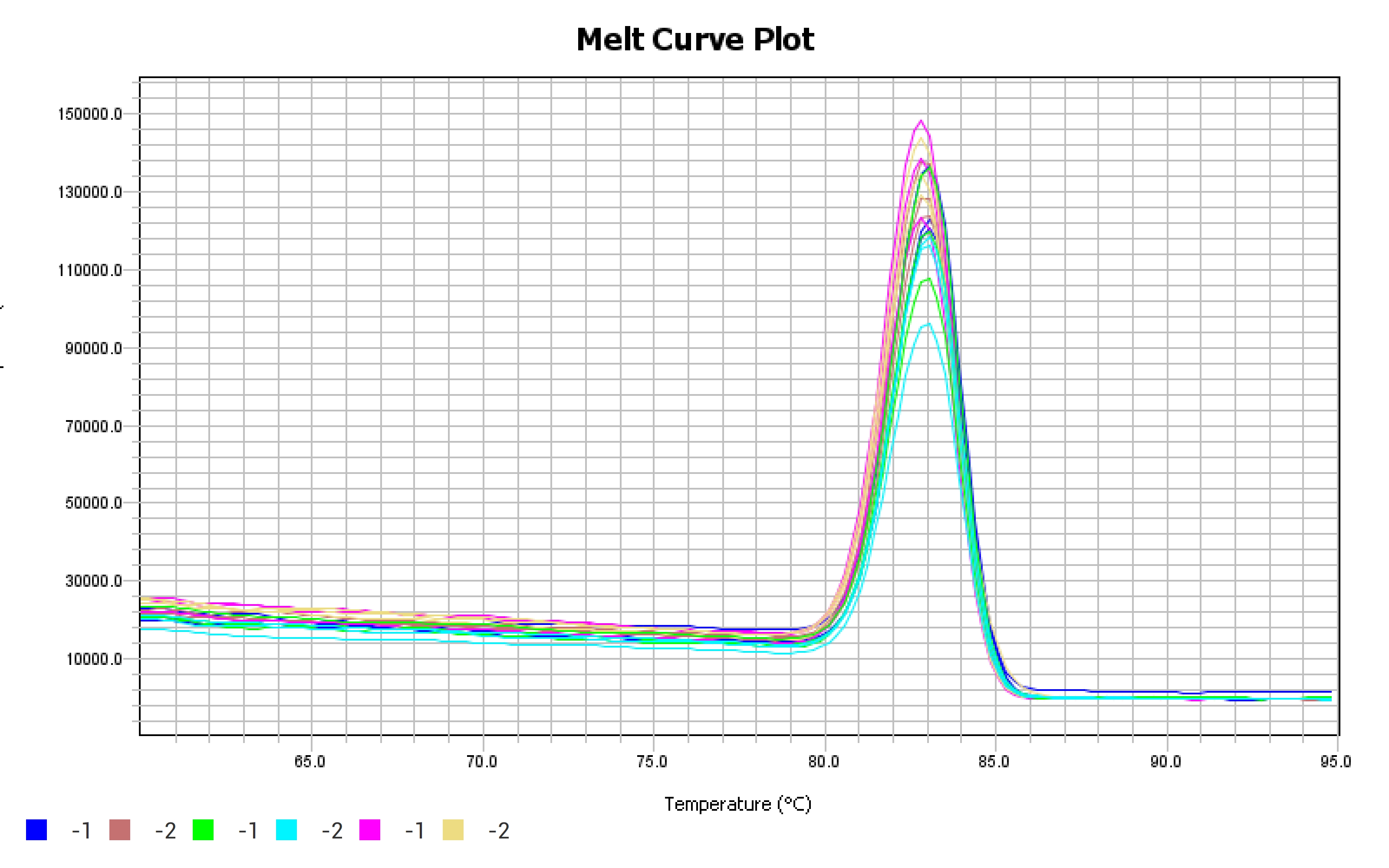

Supplement: Supplementary file 1 [file biology-14-01363-s001.zip › Figure S2-Melting Curve (for qPCR)/Melt Curve Plot-Rat1.2 TNF-a┴.jpg]

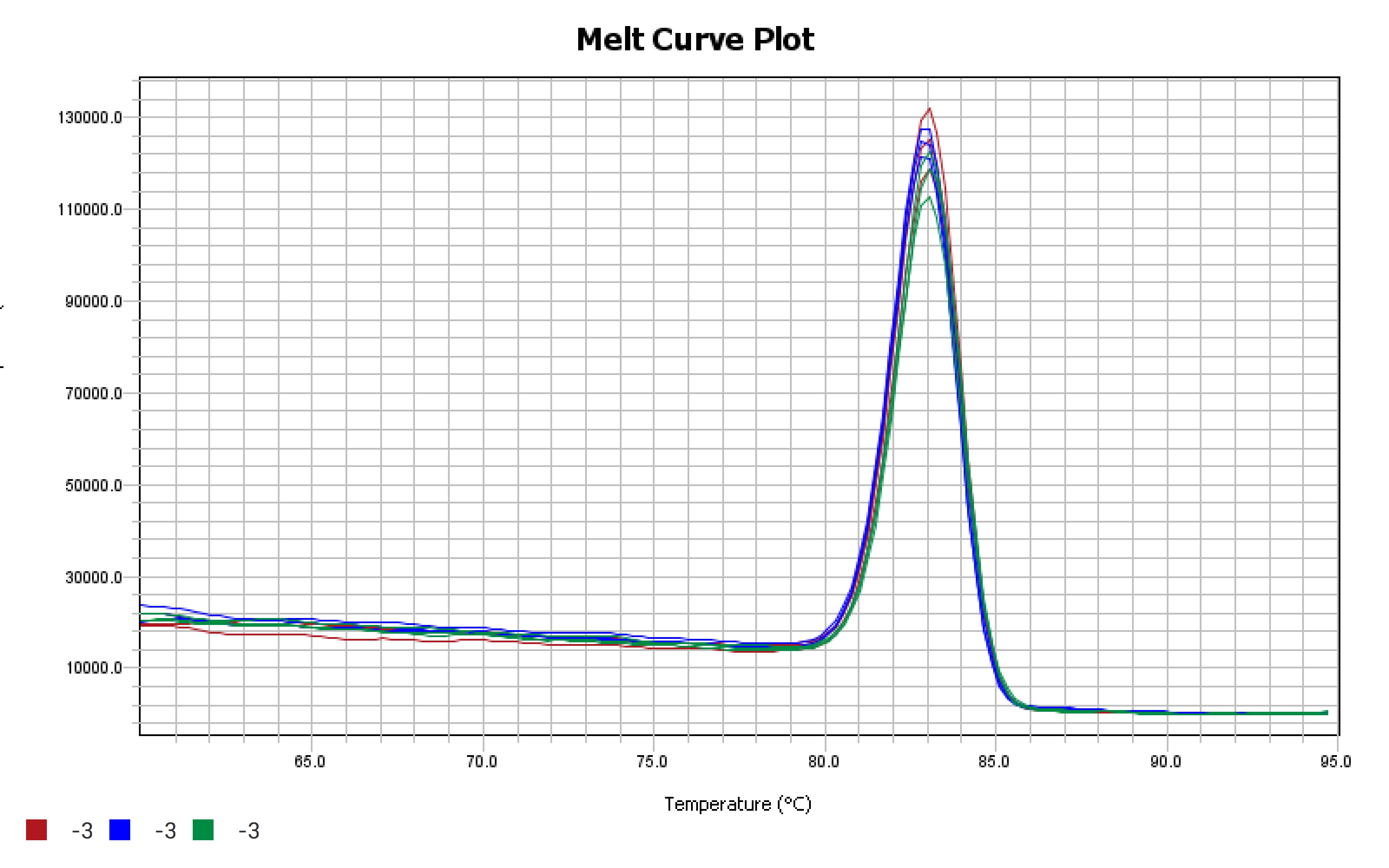

Supplement: Supplementary file 1 [file biology-14-01363-s001.zip › Figure S2-Melting Curve (for qPCR)/Melt Curve Plot-Rat3 Acta2.jpg]

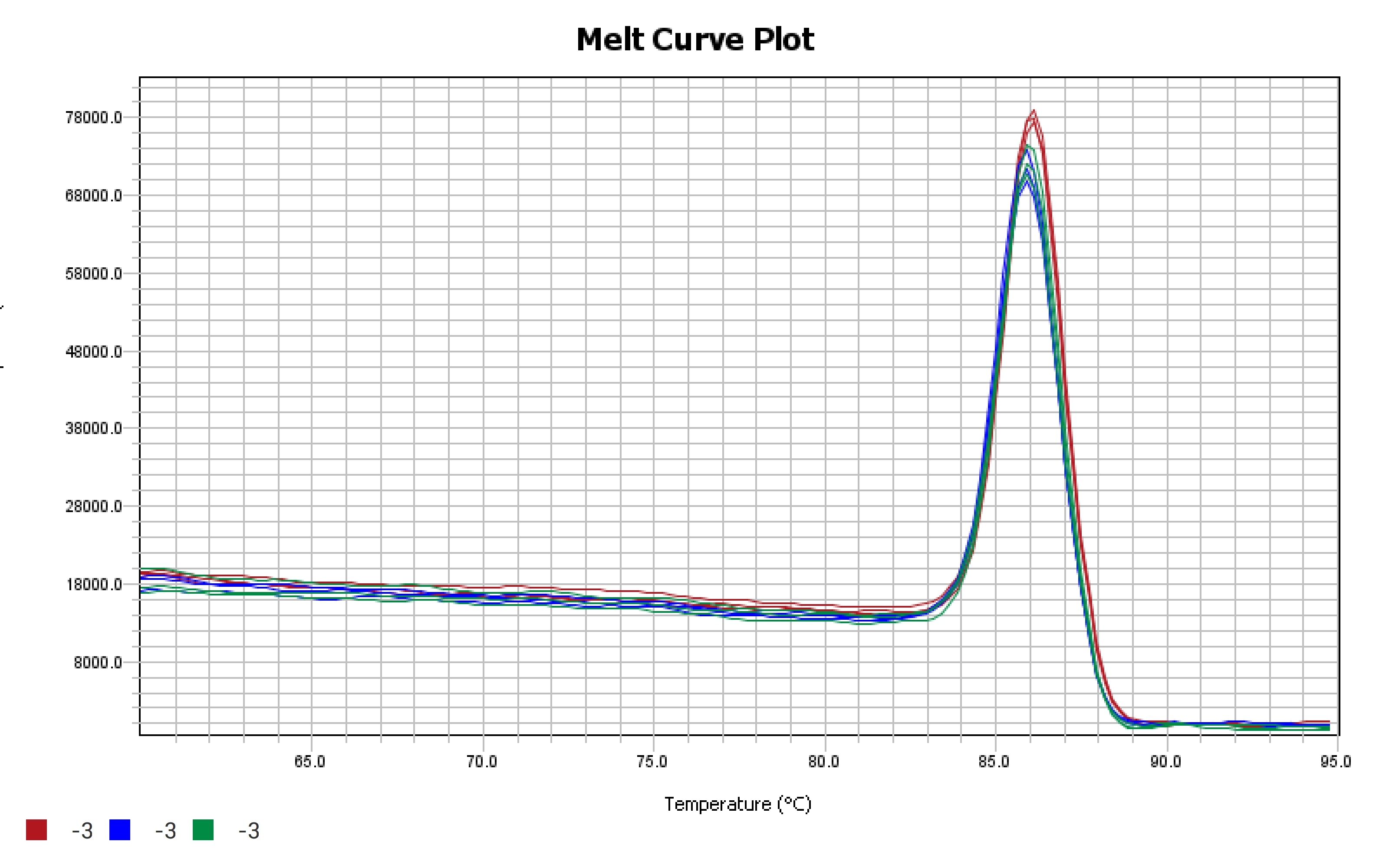

Supplement: Supplementary file 1 [file biology-14-01363-s001.zip › Figure S2-Melting Curve (for qPCR)/Melt Curve Plot-Rat3 BAX.jpg]

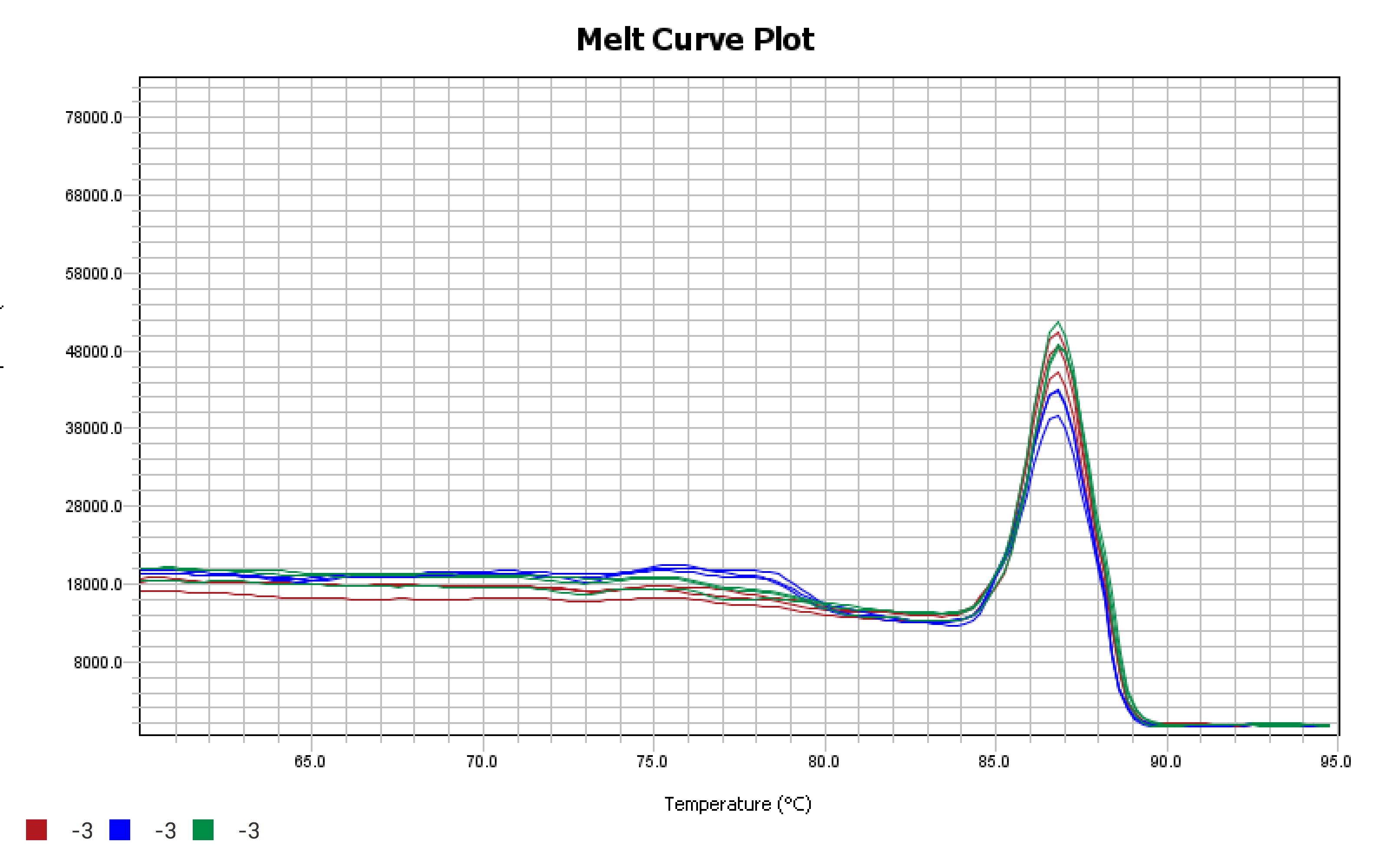

Supplement: Supplementary file 1 [file biology-14-01363-s001.zip › Figure S2-Melting Curve (for qPCR)/Melt Curve Plot-Rat3 Bcl-2.jpg]

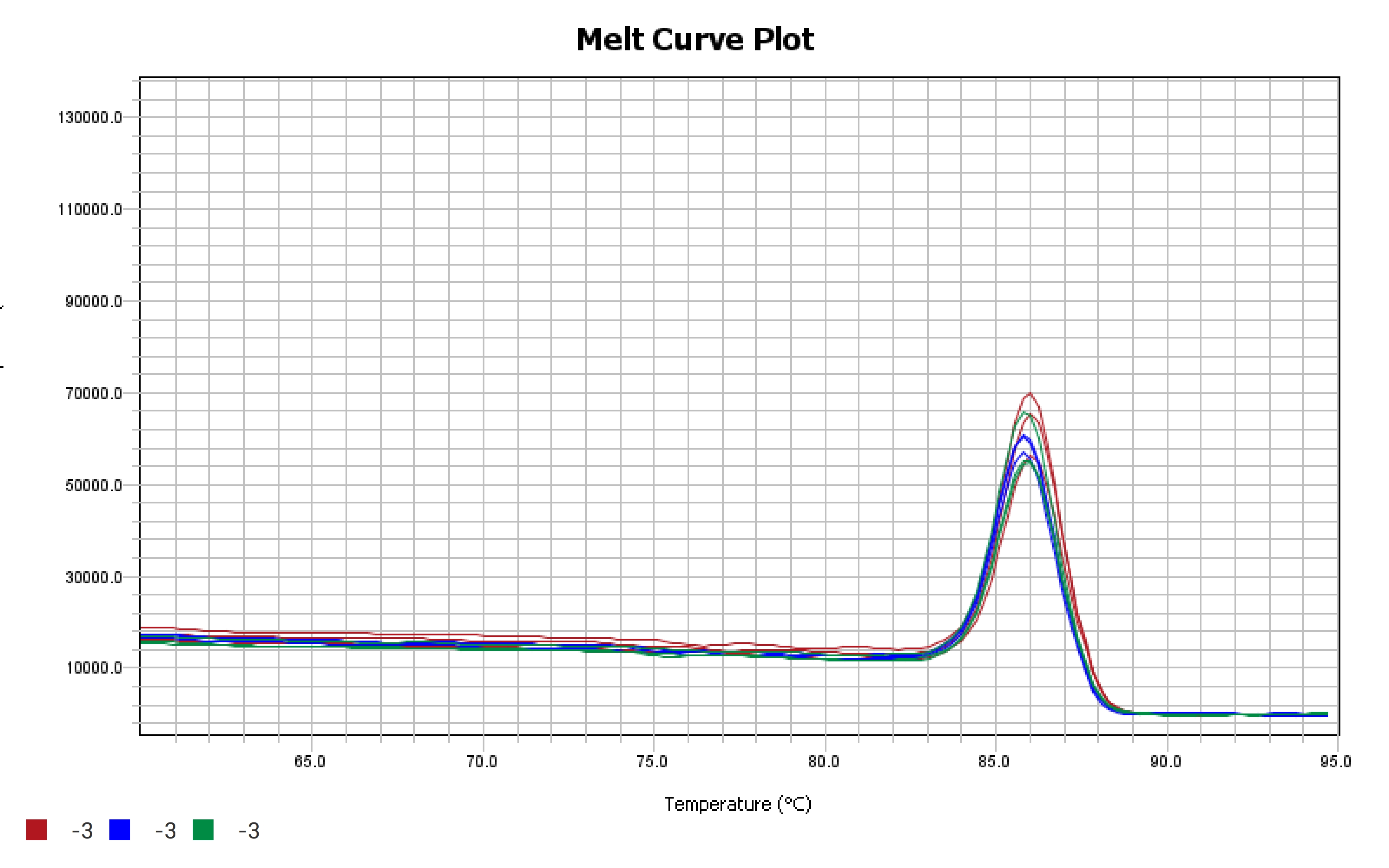

Supplement: Supplementary file 1 [file biology-14-01363-s001.zip › Figure S2-Melting Curve (for qPCR)/Melt Curve Plot-Rat3 GAPDH-1.jpg]

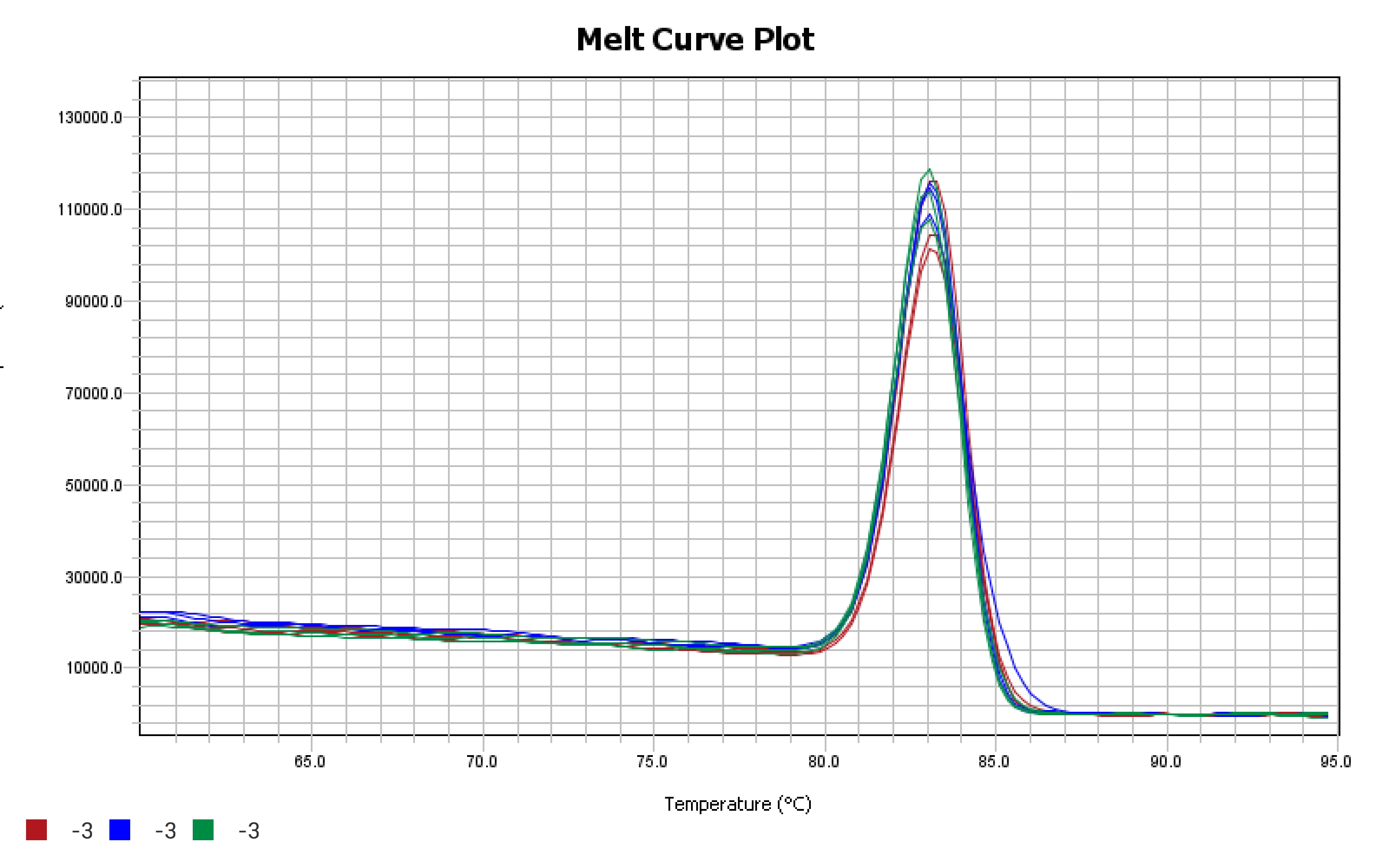

Supplement: Supplementary file 1 [file biology-14-01363-s001.zip › Figure S2-Melting Curve (for qPCR)/Melt Curve Plot-Rat3 IL-1a┬.jpg]

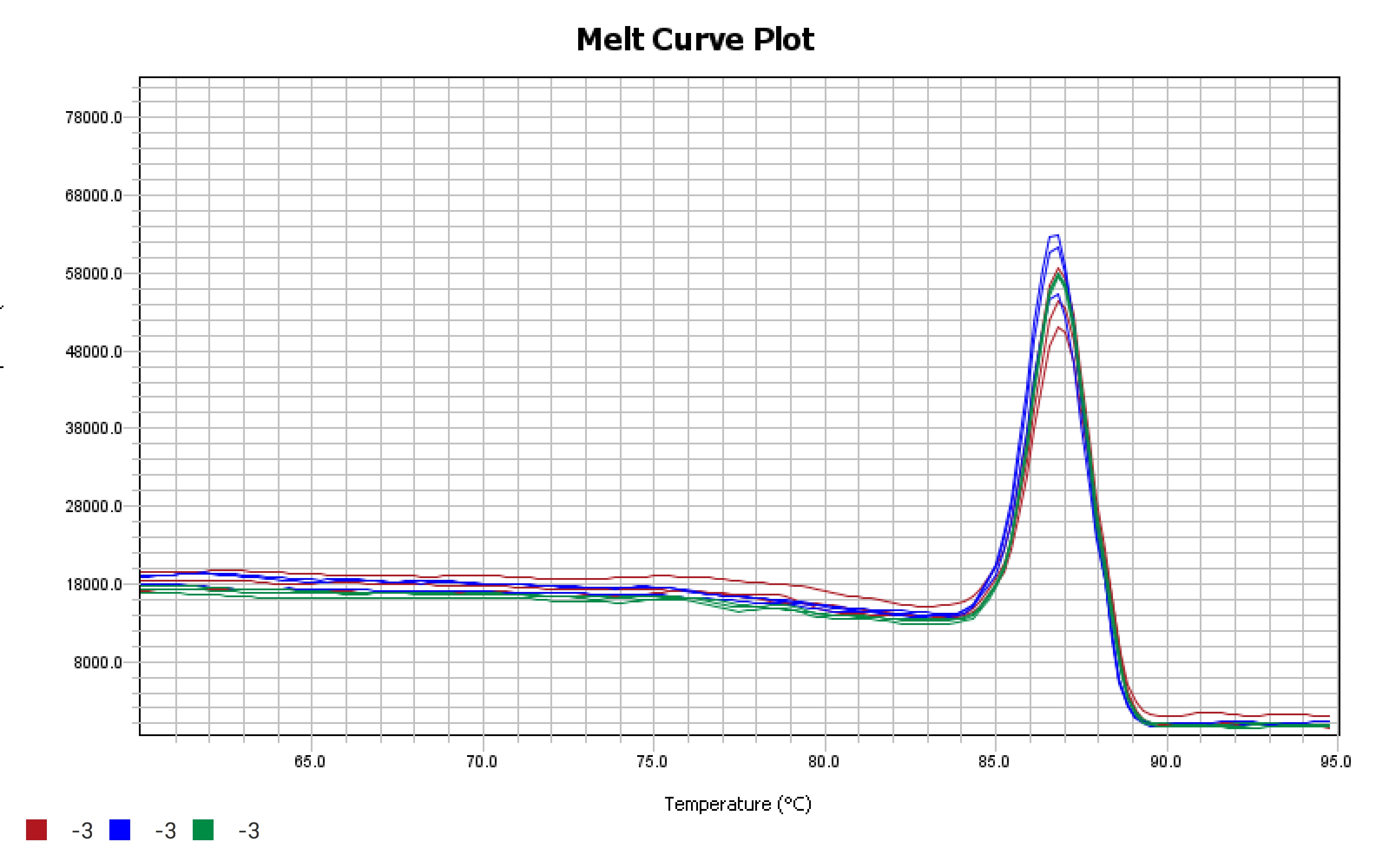

Supplement: Supplementary file 1 [file biology-14-01363-s001.zip › Figure S2-Melting Curve (for qPCR)/Melt Curve Plot-Rat3 IL-6.jpg]

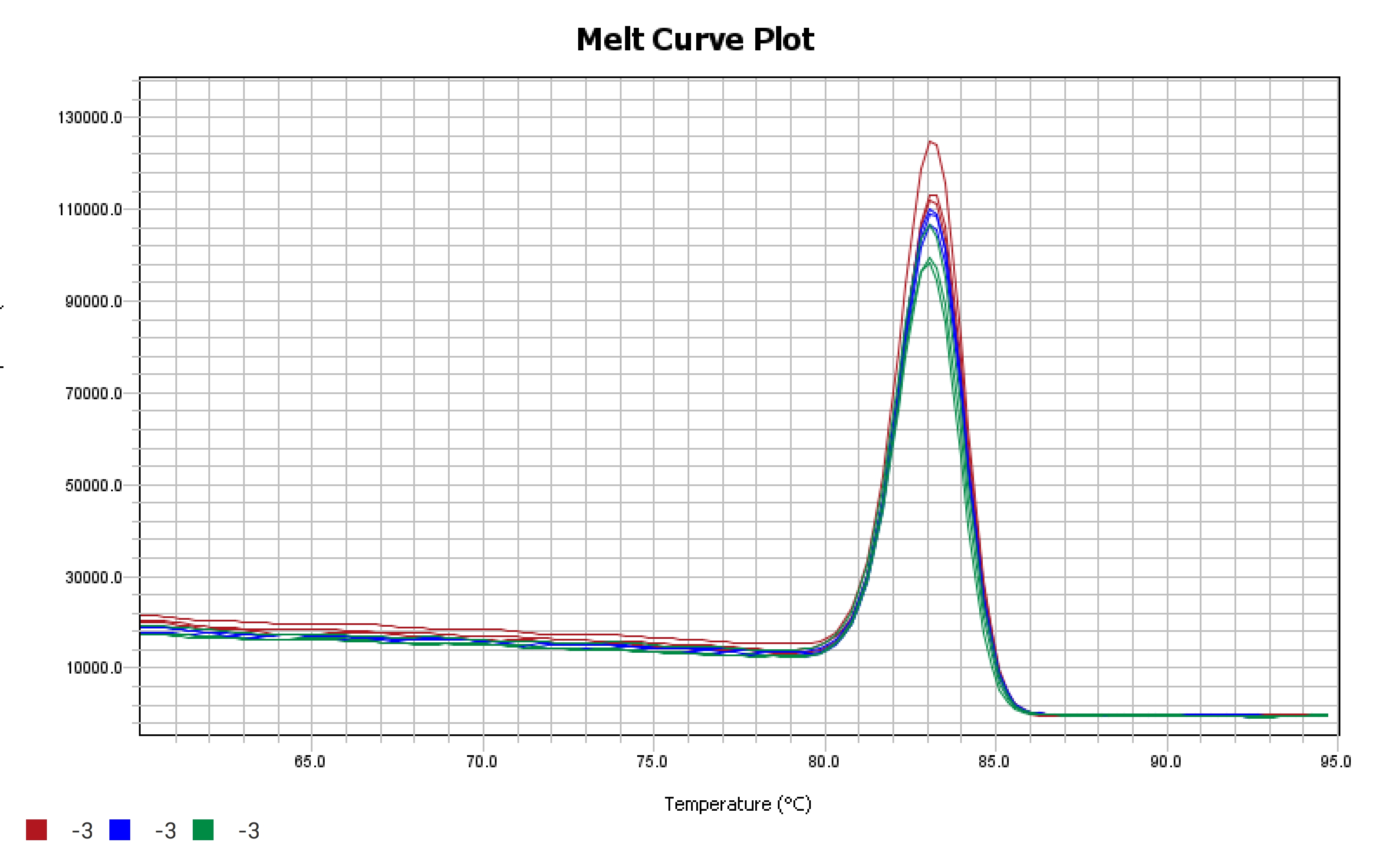

Supplement: Supplementary file 1 [file biology-14-01363-s001.zip › Figure S2-Melting Curve (for qPCR)/Melt Curve Plot-Rat3 Myc.jpg]

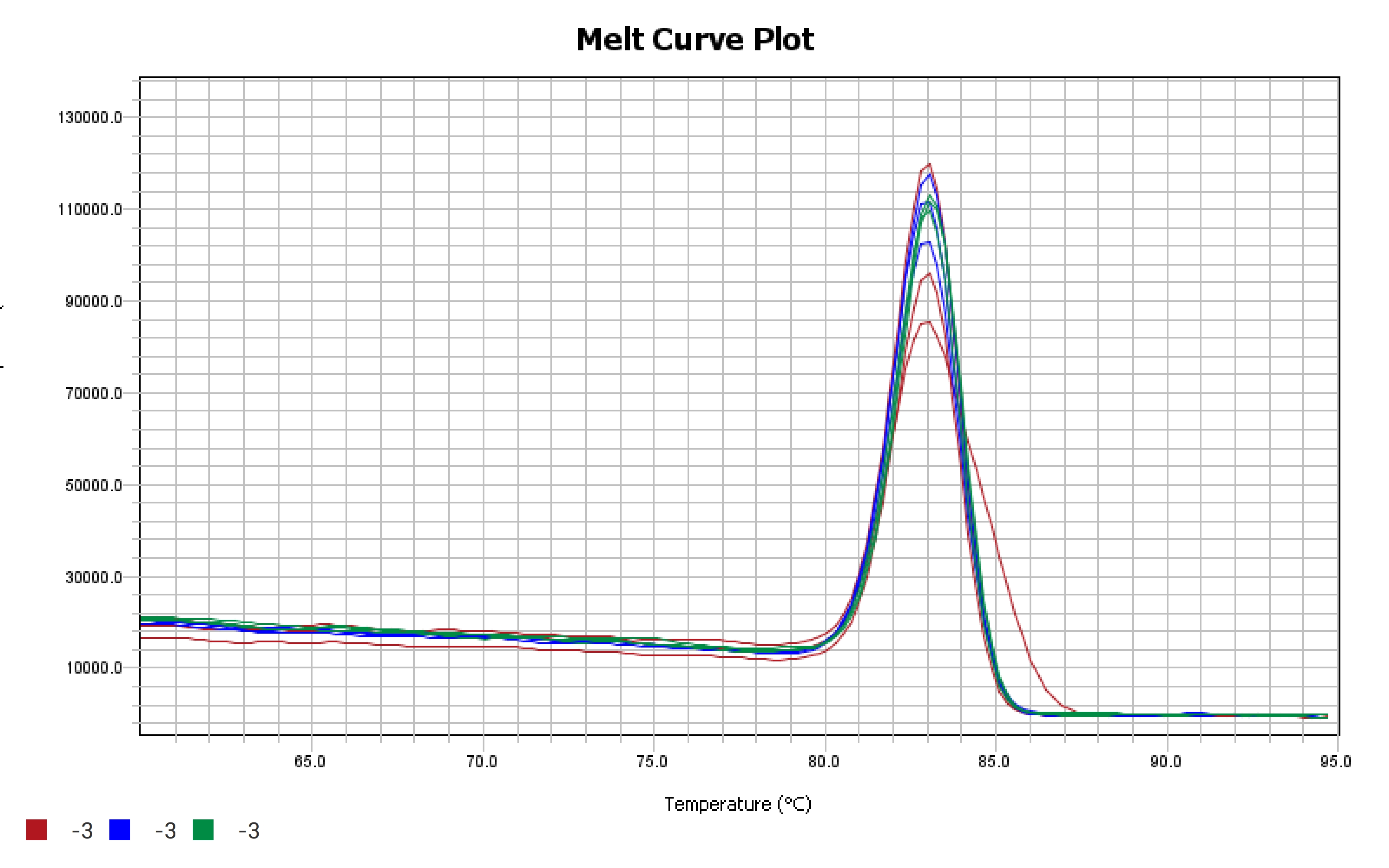

Supplement: Supplementary file 1 [file biology-14-01363-s001.zip › Figure S2-Melting Curve (for qPCR)/Melt Curve Plot-Rat3 PCNA.jpg]

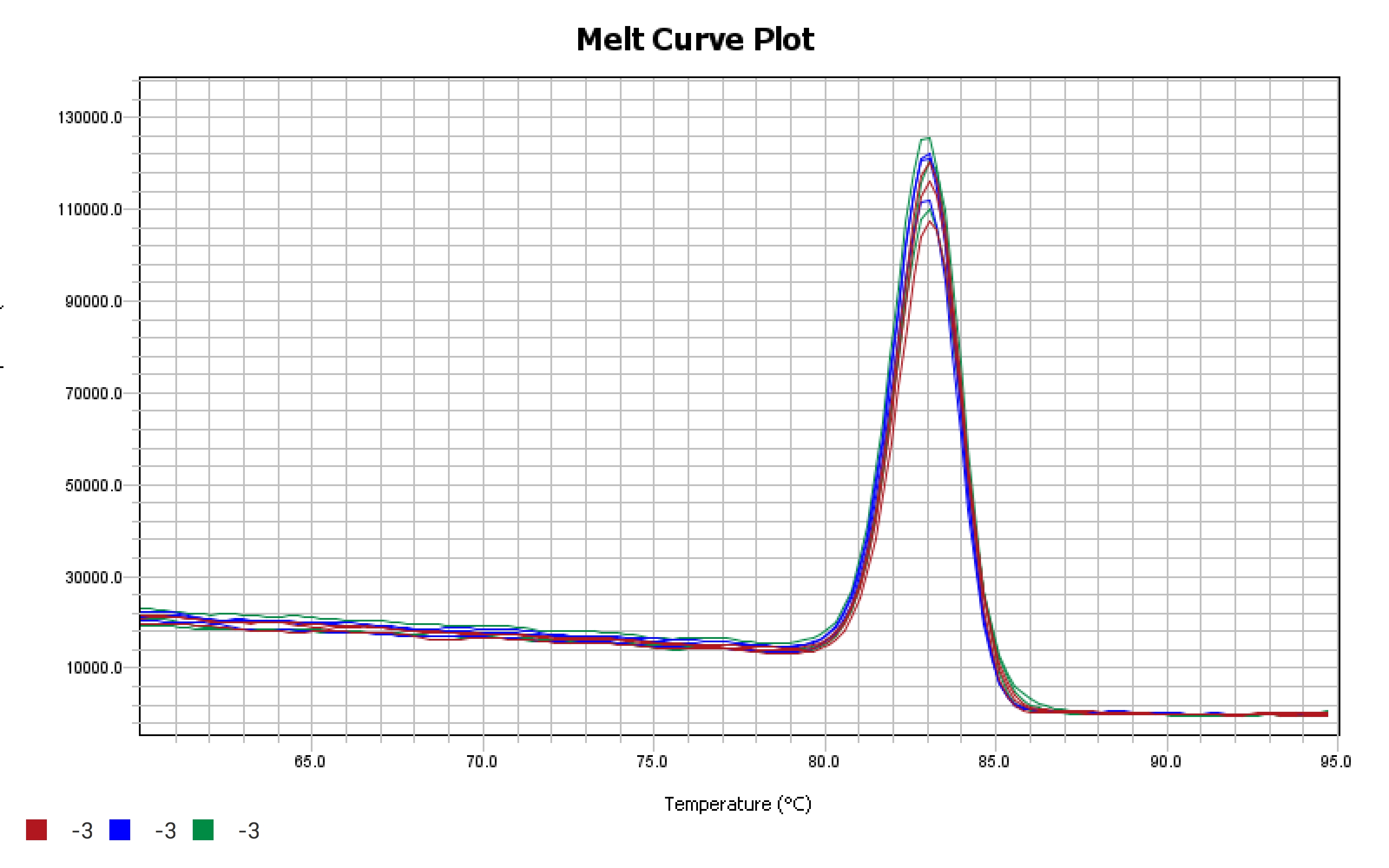

Supplement: Supplementary file 1 [file biology-14-01363-s001.zip › Figure S2-Melting Curve (for qPCR)/Melt Curve Plot-Rat3 TGF-a┬1.jpg]

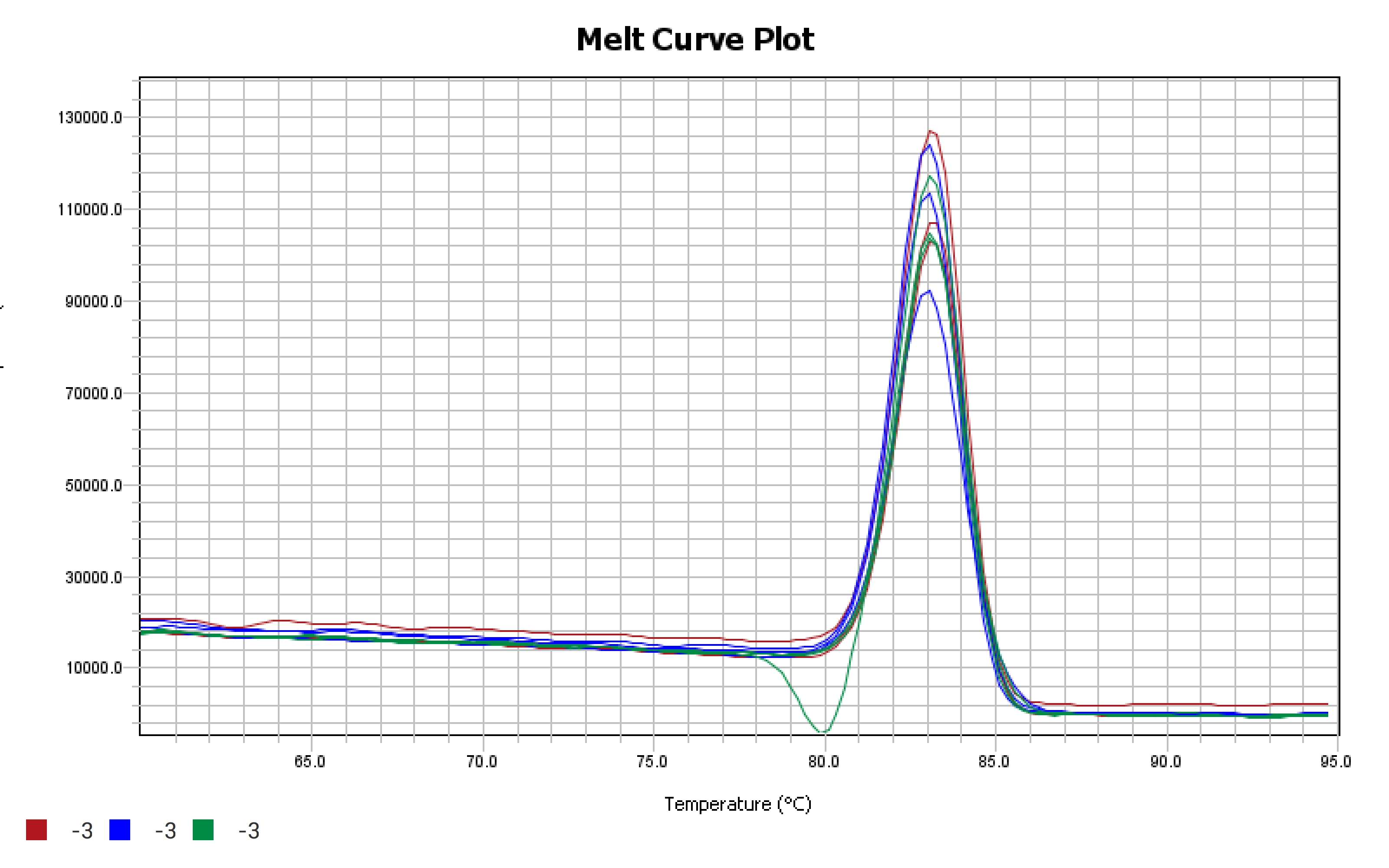

Supplement: Supplementary file 1 [file biology-14-01363-s001.zip › Figure S2-Melting Curve (for qPCR)/Melt Curve Plot-Rat3 TNF-a┴.jpg]
